# Mouse models to unravel the role of inhaled pollutants on allergic sensitization and airway inflammation

**DOI:** 10.1186/1465-9921-11-7

**Published:** 2010-01-21

**Authors:** Tania Maes, Sharen Provoost, Ellen A Lanckacker, Didier D Cataldo, Jeroen AJ Vanoirbeek, Benoit Nemery, Kurt G Tournoy, Guy F Joos

**Affiliations:** 1 Laboratory for Translational Research in Obstructive Pulmonary Diseases, Department of Respiratory Medicine, Ghent University Hospital, De Pintelaan 185, 9000 Ghent, Belgium; 2 Laboratory of Tumours and Developmental Biology, GIGA-research, University of Liège and CHU of Liège, Liège, Belgium; 3 Department of Pneumology, GIGA-research, University of Liège and CHU of Liège, Liège, Belgium; 4 Laboratory of Pneumology, Research Unit of Lung Toxicology, K.U.Leuven, Leuven, Belgium

## Abstract

Air pollutant exposure has been linked to a rise in wheezing illnesses. Clinical data highlight that exposure to mainstream tobacco smoke (MS) and environmental tobacco smoke (ETS) as well as exposure to diesel exhaust particles (DEP) could promote allergic sensitization or aggravate symptoms of asthma, suggesting a role for these inhaled pollutants in the pathogenesis of asthma. Mouse models are a valuable tool to study the potential effects of these pollutants in the pathogenesis of asthma, with the opportunity to investigate their impact during processes leading to sensitization, acute inflammation and chronic disease. Mice allow us to perform mechanistic studies and to evaluate the importance of specific cell types in asthma pathogenesis. In this review, the major clinical effects of tobacco smoke and diesel exhaust exposure regarding to asthma development and progression are described. Clinical data are compared with findings from murine models of asthma and inhalable pollutant exposure. Moreover, the potential mechanisms by which both pollutants could aggravate asthma are discussed.

## Introduction

Asthma is a chronic inflammatory disorder of the airways. The clinical hallmark of asthma is bronchial hyperresponsiveness with recurrent episodes of wheezing, breathlessness, chest tightness and cough. These episodes are associated with variable airflow obstruction that is at least partially reversible [1].

Asthma is a considerable public health concern, with an increasing prevalence and an estimate of 300 million asthmatics worldwide. Although the cause of asthma is unknown, there are several risk factors that influence the development of asthma. These can be divided into host factors and environmental risk factors [1]. The allelic distribution of genes pre-disposing to atopy or airway hyperresponsivess is a typical *host factor *which determines asthma development and phenotype. Typical *environmental factors *are allergens (indoor or outdoor allergens, such as these originating from domestic mites, furred animals, cockroach, fungi, molds, yeasts and pollen), infections (mainly viruses), occupational sensitizers, tobacco smoke (both active and passive smoking) and indoor or outdoor pollution by gasses and particulate matter (PM) [1,2].

In their efforts to unravel the pathogenesis of asthma, researchers have mainly focussed on the basic immunologic mechanisms resulting in unwanted or exaggerated inflammation. Many uncertainties remain concerning why and how asthma develops during lifetime. The emerging hypothesis is that a failure of endogenous immune regulated tolerance mechanisms might be involved [3]. Alternatively, exposure to a more or less specific cocktail of allergens or pollutants might also lead to the development of an asthmatic phenotype [2]. Regardless of the mechanism, exposure of the airways to foreign agents (allergens or chemical agents) often represents the very first cause for an immune derailment. In later stages, sensitized individuals will be more susceptible to develop airway inflammation and symptoms. These processes can be present for a limited time or become chronic. In that view, the pathogenesis of allergic asthma comprises 3 phases: sensitization, acute inflammation and chronic disease.

The association between exposure to inhalable pollutants such as cigarette smoke and PM (e.g. diesel exhaust) and respiratory morbidity has been recognized for a long time. The epidemiological association of increased exposure to air pollutants and the rise in frequency of wheezing illnesses led to the assumption that these pollutants are actively involved in the pathogenesis of asthma. While there is no doubt that inhaled pollutants can exacerbate the symptoms of asthma, it is also considerable (though less well established) that they play a role in inducing asthma or at least in driving incipient asthma into clinically obvious manifestations of the disease.

**Figure 1:**
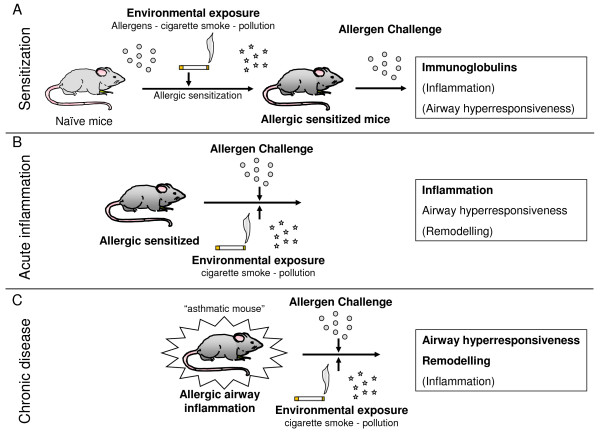
**Schematic presentation of how the effects of environmental exposures, (aero)immunization and airway challenge on the 3 different phases of the asthma pathology (sensitization, acute inflammation and chronic disease) can be dissected in mice**.

A widely used tool to evaluate the effects of inhaled pollutants on the development and aggravation of asthma consists in epidemiological studies. Controlled exposure studies in humans are informative as well, but are limited by practical and ethical issues. The use of animal models leads to more insights regarding the role of inhalable pollutants during sensitization and inflammation in asthma, with a unique opportunity to unravel the effects on the different phases of the development of the asthma pathology (Figure 1). The mouse has emerged as the animal of choice for modeling this disease [4]. In this review, we give a summary of the studies investigating the impact of inhaled pollutants on the onset, development or aggravation of asthma. We particularly focussed on tobacco smoke and PM, more specifically diesel exhaust particles (DEP).

## Health effects of tobacco smoke and diesel exhaust particles

The World Health Organization (WHO) reports 1.15 billion smokers, of whom 200 million live in Europe [5]. The yearly production of cigarettes still increases in order to meet the people's wishes. Tobacco smoke is a complex mixture of more than 4000 components [6-8]. Researchers distinguish two different emissions from cigarettes. Mainstream smoke (MS) is the smoke actually inhaled by the cigarette smoker (active smoking), whereas side stream smoke is released from the burning end of the cigarette. In many epidemiologic studies, the term environmental tobacco smoke (ETS) is used, which is a mixture of sidestream smoke and exhaled mainstream smoke in the environment after dilution and aging. This mixed smoke is inhaled during passive smoking (also referred to as "second hand smoke"). Active and passive smoking contribute to the development of various respiratory health problems such as asthma and reduced lung function. In susceptible individuals, active smoking is associated with structural changes in the airways (remodelling, especially of the small airways) and results in destruction of the lung parenchyma (emphysema) [9,10]. Chronic exposure to cigarette smoke induces clinically significant chronic obstructive pulmonary disease (COPD) in 20% of the smokers. Besides COPD, tobacco smoking also causes lung cancer and other adverse health effects [5].

The WHO reports that there is consistent evidence that airborne particulate matter (PM) has a measurable public health impact [11]. The range of health effects is broad, but affects predominantly respiratory and cardiovascular systems. All population is affected, albeit susceptibility to the pollution may vary with age or health status. The risk for various outcomes increases with exposure and adverse effects of PM were demonstrated after both short-term and long-term exposure [11]. Diesel exhaust particles (DEP) are an important component in ambient air pollution and respirable particulate matter. They consist of a carbon core and adsorbed organic substances such as polycyclic aromatic hydrocarbons, and contain small amounts of sulphate, nitrate, metals and other trace elements [12,13]. The majority of diesel exhaust particles are ultrafine particles with a diameter around 0.1 μm, that are highly respirable, reaching the alveoli and the systemic circulation [14]. DEP exposure can induce acute irritation to eyes and throat, light-headedness and nausea, and has been associated with increased respiratory symptoms (cough, phlegm, chronic bronchitis, asthma), increased lung cancer risk and increased risk for total mortality and cardiopulmonary mortality [13-15].

## Clinical data on the effect of inhaled pollutants on allergic sensitization and asthma

It is generally accepted that sensitization to allergens is a crucial risk factor for the development of asthma. Studying the potential effects of environmental factors on allergic sensitization is thus relevant. Although the nature of the inhaled particles from cigarette smoke and DEP differs, they induce a similar inflammatory response which is characterized by neutrophils, T-lymphocytes, increased levels of IL-8 and IL-6, along with a decreased phagocytic capacity of alveolar macrophages [12,14,16-19]. One can hypothesize that both cigarette smoke and DEP deploy similar mechanisms, creating an environment which facilitates allergic sensitization and asthma development.

Epidemiological studies cannot provide the proof that cigarette smoke or DEP exposure are causative factors in the development of asthma. However, associations between the risk of developing asthma and inhalable pollutant exposure provide strong indications that there might be a causal relationship. Many data support the hypothesis that **ETS **exposure (passive smoking) contributes to the development of both childhood asthma and adult onset asthma [20]. *In utero *exposure to maternal smoking or any smoking at home significantly increases the risk for developing childhood asthma [21,22]. The prevalence of asthma, wheezing, chronic cough and breathlessness in children increases with the number of parents smoking, suggesting a dose-response [23]. ETS exposure during childhood [24] or in adults - mainly occupational exposure - is also associated with the development of adult asthma and other respiratory symptoms [25] in a dose-dependent manner [26]. However, the relationship between ETS exposure and allergic sensitization (as evaluated by serum IgE and skin prick tests) is less evident [27,28]. ETS can promote the induction of Th2 cytokines in nasal fluid of allergic patients, indicative of allergic response exacerbation by ETS in human beings [29]. Passive smoking is indeed dose-dependently related to greater asthma severity, diminished pulmonary function and poorer asthma control in adults [30,31] as well as children [32,33]. The correlation between ETS and asthma prevalence and severity is extensively reviewed in [34].

Contrasting with ETS, the impact of active smoking on the development of asthma is more controversial. Some reports state that active smoking is not a risk factor for adult onset asthma [35], whereas other reports demonstrate the opposite [36,37]. Active smoking during adolescence increases the risk of new onset asthma [38,39]. Asthma prevalence is also higher both in adolescents [40] and among the elderly [41] who smoke. Current wheezing, current asthma and lifetime asthma are all related to active smoking [42]. As for passive smoking, the relationship between active smoking and atopy is again controversial [43,44]. Active smoking is associated with asthma severity, with higher asthma severity scores [35] and less controlled asthma [45]. Smoking asthmatics have a reduced lung function [46], greater decline in FEV1 with age [47] and the lung function is inversely correlated with the amount of cigarettes smoked per day [48]. Active smoking in asthmatics also impairs the therapeutic response to corticosteroids [49].

Many epidemiological data suggest that traffic related air pollution (rather than DEP as such) is a risk factor for wheezing, asthma prevalence and allergic sensitization (reviewed in [50-52]), however the evidence is not so strong. Nevertheless, several recent birth cohorts demonstrated positive correlations between exposure to traffic pollution and atopic diseases and allergic sensitization in children [53,54]. In contrast to the epidemiological knowledge, experimental approaches have convincingly demonstrated that DEP can facilitate the induction of allergic sensitization. Besides their ability to increase in vivo IgE and cytokine production at the upper respiratory mucosa, DEP can facilitate sensitization to a neoallergen, with the production of allergen-specific IgE and skewing of cytokine production to a T-helper cell 2 pattern [15,55,56]. Ambient air pollution is associated with asthma severity [34,57,58], but reported effects of DEP on aggravation of asthma in controlled exposure studies differ, possibly due to the variety of exposure regimens used in experimental protocols [17,59]. A recent crossover study in London in mild to moderate asthmatics with real life exposure to diesel traffic demonstrated an asymptomatic, though significant reduction in lung function (FEV_1 _and FVC), most pronounced in the moderate asthmatics and accompanied by increases in inflammatory markers [60].

## Inhaled pollutants and murine allergic sensitization

Mouse models of asthma allow analyses in precisely defined environmental conditions. A commonly used experimental allergen in mouse models is the inert protein ovalbumin (OVA), but also house dust mite, pollen and *Aspergillus *models exist. Sensitization towards OVA, either naturally or upon inhalational exposure, does generally not occur in mice. On the contrary, mice develop inhalational tolerance and become refractory to subsequent immunization attempts by OVA intraperitoneally [61,62]. Some studies intend to break inhalational tolerance by combined exposure regimens in the absence of any intraperitoneal injection, whereas other studies examine the aggravating or modulating effects of inhaled pollutants on the sensitization phase in previously sensitized animals (Figure 1). The impact of tobacco smoke or DEP on allergic sensitization or inflammation in different mouse asthma models will be discussed. In cigarette smoke exposure models, both nose-only and whole body exposures are performed. Side stream smoke is often used as a surrogate for ETS and will be referred to as ETS hereafter. DE(P) models use intranasal or intratracheal DEP-applications or diesel exhaust (DE) inhalation. Additional files 1, 2 and 3 give a detailed overview of methodologies and results from studies with ETS, MS and DEP respectively.

### Cigarette smoking and sensitization in mice (Additional files 1 and 2)

Rumold and colleagues proved that **ETS (passive smoking) **can act as an adjuvant for allergic sensitization to OVA [63] (Additional file 1). The co-exposure to ETS and aerosolized OVA induced de novo sensitization, with the development of a memory response [63]. ETS also enhanced allergic sensitization towards intraperitoneal OVA and the effects of ETS were more profound in females, compared to male mice [64,65]. However, in other reports the effects were less clear [66,67]. For example, chronic postnatal exposure to combination of OVA and ETS tended to reduce OVA-specific immunoglobulin production compared to OVA-alone exposure and showed no evident effects on pulmonary inflammation, although airway hyperresponsiveness was increased. Accordingly, ETS-exposure prior to and concomitant with OVA-aerosol exposure could not overcome airway tolerance in three different mouse strains with a different level of susceptibility to airway hyperresponsiveness (A/J, BALB/c and C57BL/6) [67]. Also *in utero *exposure to ETS did not affect antibody production or airway inflammation towards postnatal aerosolized OVA in unsensitized animals, although it did increase airway hyperresponsiveness [68]. ETS exposure prior to, during and after several intranasal sensitizations towards another allergen, *Aspergillus fumigatus *(Af), did not affect IgE-production, but it did increase blood eosinophilia and airway hyperresponsiveness [69]. Thus, despite the absence of IgE markers of sensitization, ETS repeatedly aggravated hyperresponsiveness in different models.

Not all reports are univocal, but some murine models support the hypothesis that ETS can behave as an adjuvant and facilitate allergic sensitization. Although not yet proven, facilitation of allergic sensitization could explain the reported associations between ETS and the increased risk for developing asthma in humans.

In a model mimicking active smoking, in which mice were first exposed for 2-3 months to mainstream cigarette smoke (MS) and subsequently sensitized to OVA or ragweed via the mucosa, smoke exposure increased Th2-cytokine production by splenocytes (suggestive for a heightened allergic sensitization), but attenuated pulmonary inflammation and airway hyperresponsiveness [70] (Additional file 2). In another model without intraperitoneal sensitization, MS could disrupt the normal tolerogenic immune response towards OVA [71]. While OVA aerosol could not induce *per se *any allergic inflammation, simultaneous exposure to OVA and MS induced OVA-specific IgE and IgG_1_, pulmonary inflammation and goblet cell hyperplasia [71,72]. In a similar experimental setting, concurrent exposure to MS and OVA induced allergic sensitization with antigen-specific memory in a GM-CSF dependent fashion [73]. However, prolonged cigarette smoke exposure suppressed eosinophilic inflammation in this model, indicating that cigarette smoke potentially bears both adjuvant and anti-inflammatory properties.

All reports with MS suggest that active smoking can facilitate sensitization in mice, however the data on the subsequent development of allergic inflammation in mice are contradictory. This underscores the need to further elucidate the impact of experimental conditions, which can favour inflammation or, on the contrary, suppress immunity, probably depending on the dose, method and duration of cigarette smoke exposure. Considering that the impact of active smoking on the development of asthma is controversial, these mouse models are very challenging and merit further investigation.

### Diesel exhaust particle exposure and sensitization in mice (Additional file 3)

Muranaka and coworkers were the first to show that DEP can increase specific IgE towards OVA or Japanese Cedar Pollen (JCP) after intraperitoneal sensitization [74,75] (Additional file 3). Since then, many authors have described the adjuvant effects of DEP, using different immunization routes. DEP or diesel exhaust can increase OVA-specific IgE, and can increase IL-4 production and cell proliferation in mediastinal and cervical lymph nodes or spleen after intratracheal, intranasal and inhalational sensitization, respectively [76-78]. DEP can thus affect the antigen-specific IgE antibody responses through local and systemic T-cell activation. Similar observations were reported upon sensitization through injection into the footpad [79]. Both the organic matter adsorbed to DEP and the non-extractable carbon core are thought to be responsible for the adjuvant effect [79-81]. Several sensitization models using OVA or house dust mite (Der f) in presence of DEP revealed also increased antigen-specific IgG1 and IgG2 levels, besides increased antigen-specific IgE [81-88]. Moreover, DEP aggravates the observed pulmonary inflammation and goblet cell proliferation in these models.

In line with the experimental data in humans, DE or DEP (self-produced or commercially available reference material) have consistently shown to facilitate allergic sensitization. In contrast to the above mentioned effects of tobacco smoke, biological effects of DEP in mice seem to be less affected by experimental conditions.

## Inhaled pollutants and allergen-induced murine asthma models

Different approaches can be used to evaluate the effects of inhaled pollutants on the pathogenesis of allergen-induced airway inflammation.

Firstly, experimental models can evaluate the effect on asthma development. Animals are challenged with allergen in the presence of inhalable pollutants and develop a typical asthmatic phenotype (IgE, pulmonary inflammation, T-cell responses, airway hyperresponsiveness, goblet cell hyperplasia and remodelling) (Figure 1B). The timepoint where the inhalable pollutant is introduced can vary: (1) before the first allergen challenge: assuming that an alteration of the pulmonary environment might induce a higher sensitivity to subsequent allergen challenge; (2) simultaneous exposure: assuming that the presence of the inhalable pollutant and the allergen can affect the pulmonary response to both agents, and in which a possible interaction between both agents can become relevant.

Secondly, models can also evaluate the aggravating effects of inhalable pollutants on mice with previously established allergic airway inflammation, reflecting the human situation of pollutant exposure in existing asthma (Figure 1C).

Additional files 4, 5 and 6 give a detailed overview of methodologies and reported observations in mouse models in which the effects of ETS, MS or DEP exposure on the development or aggravation of asthma were examined.

### Cigarette smoking and development or aggravation of asthma in mouse models (Additional files 4 and 5)

Different in vivo studies have demonstrated that ETS can aggravate the allergic response in mice which were primed with OVA and had already mounted a Th2 response. Indeed, ETS exposure prior to and during allergen challenge in sensitized mice induces an upregulation of the allergic response, with increased systemic and pulmonary inflammation, which is more pronounced in females compared to males [64,65] (Figure 1B) (Additional file 4). In this experimental setup, the mice, however, also exhibited heightened allergic sensitization (see section on ETS and sensitization), hampering the distinction between effects on sensitization, on asthma development or on both. Enhanced pulmonary inflammation, remodelling and hyperresponsiveness were also observed upon chronic co-exposure to ETS and OVA in "asthmatic mice" (Figure 1C) [89]. *In utero *exposure to ETS has long term effects on the development of allergic inflammation and exacerbates subsequent adult responses to initial allergen exposure [68]. Maternal smoking during pregnancy also induces airway remodelling in mice offspring [90].

In contrast to the reports on ETS, the effects of mainstream cigarette smoke (MS) on the development and exacerbation of allergic inflammation in mice are a matter of debate [91-93] (Additional file 5). Some authors reported that MS exposure inhibits OVA-induced airway hyperresponsiveness and reduces inflammation in a model of established asthma [70,92]. However, in a BALB/c model examining the development of allergic inflammation, Moerloose et al [93] demonstrated that acute concurrent exposure to allergen (OVA) and MS enhances the allergic pulmonary inflammation, and augments OVA-specific IgE production and airway hyperresponsiveness [93]. These acute effects were confirmed in C57/Bl6 mice [94]. Upon prolonged exposures, however, the combination OVA/smoke could delay - though not prevent - the development of tolerance, which is classically observed upon chronic OVA-aerosol exposures [94]. In an "asthmatic mouse", chronic co-exposure to MS and OVA did neither aggravate airway inflammation, OVA-specific IgE production and remodelling, nor accelerate emphysema development [95]. Interestingly, smoke exposure did increase OVA-specific IgE levels in sensitized mice, suggesting that atopic smokers may be at risk for increased allergen-specific IgE, thus increasing their risk for developing asthma [95]. Recently, the importance of the smoke exposure regimens was highlighted, since high dose, but not low dose MS suppressed allergic airway inflammation by inhibiting T-cell function [96].

### Complexity of cigarette smoke exposure models

Although is generally accepted that both active and passive smoking aggravate the severity of asthma in man, murine models suggest the relationship is not that simple. In murine asthma models, there is a discrepancy in the effects of ETS and MS. ETS consistently aggravated all measured outcomes in murine models, similar to observations in humans. MS however, aggravated the development of allergic asthma on the one hand, but it could also suppress established allergic inflammation on the other hand. The origin of the discrepancy between ETS and MS is difficult to define, but can relate to differences in the dose, chemical composition or even particle size of ETS vs MS. Mimicking active smoking in mice is a challenging task and is possibly more subject to variation than ETS exposure. Since mice not "just light a cigarette and smoke", they receive MS by whole body exposure or nose-only exposure. Besides the dose, carbon monoxide levels and stress by the experimental environment (exposure in group vs. individual restrainers) could conceivably impact the immunological response. High doses of cigarette smoke could suppress T-cell or dendritic cell function or induce an increase of blood carboxyhemoglobin levels, which may have immunosuppressive effects on the ensuing allergic inflammation [96,97], whereas low doses of cigarette smoke might promote allergic inflammation. The complexity of effects induced by tobacco smoke exposure is due to its multipartite nature. Immunosuppressive and anti-inflammatory effects of tobacco smoke are mediated by its oxidants, by carbon monoxide, nicotine and some aromatic compounds that modify transcriptional programmes [98]. Cigarette smoke can moreover chemically modify signalling pathways and extracellular matrix through acetylation, nitrosylation, carbonylation and oxidation which affects cell survival, activation and differentiation [98]. On one hand, smoke exposure can lead to chronic inflammation and damaged respiratory epithelium. On the other hand, tobacco smoking can also acutely suppress epithelial function by increasing permeability and impairing mucociliary clearance. Cigarette smoke can induce infiltration of alveolae by activated macrophages, producing pro-inflammatory mediators, reactive oxygen species and proteolytic enzymes, resulting in inflammation and tissue damage. But, it can also compromise macrophage phagocytic capacity or skew their inflammatory mediator profile [98]. This dual nature of smoke acting on biological processes as both stimulus and suppressor is probably differently reflected in each experimental system, adding to the discrepancies in the reported observations.

In addition, in chronic models with MS or ETS exposure, there is a possibility to obtain phenotypes which overlap with COPD [99-101]. The development of emphysema and airway remodeling for example, which have been reported upon chronic MS exposure [99,100,102] could affect the pulmonary function measurements in an allergic setting. Also lymphoid follicle formation which has been reported in COPD mouse models [103] could contribute to allergic sensitization. This COPD aspect adds to the complexity in interpreting the data, but it can also lead to the development of clinically relevant models of an asthma/COPD overlap syndrome. In any case, further analysis of the animal models and elucidation of the involved mechanisms could provide us with valuable tools to further unravel how tobacco smoke aggravates allergic asthma in humans.

### DEP and development or aggravation of asthma in mouse models (Additional file 6)

Besides their effects on allergic sensitization, diesel exhaust particles can enhance the allergen-induced airway inflammation. In most studies, animals are exposed to DEP or diesel exhaust throughout both periods of sensitization and allergen challenge, which renders it difficult to dissect effects on either sensitization or developing airway inflammation. Most reports, however, show that both intratracheal instillation of DEP and inhalation of diesel exhaust increase the allergic response towards OVA or house dust mite in a dose-dependent way with enhanced pulmonary infiltration and local cytokine production, increased goblet cell hyperplasia, increased airway hyperresponsiveness and, in some strains, increased levels of allergen-specific immunoglobulins [104-112] (Additional file 6).

The aggravating effect of DEP on pre-existent asthma has been examined by exposing previously sensitized and allergen-challenged "asthmatic" mice to DE(P) without further exposure to allergen. In two different models, DE and DEP-exposure clearly increased airway hyperresponsiveness [113,114], but the effects rapidly subsided with continued DE-exposure [113]. The impact on pulmonary inflammation was, however, less pronounced, with no effects of DE(P) on BAL cell numbers and limited effects in the lung.

The above mentioned reports demonstrate that DEP facilitate allergic inflammation and aggravate airway hyperresponsiveness in murine models and correspond with epidemiological data associating particulate air pollution with asthma severity.

## Effects of inhaled pollutants in other animal asthma models

In guinea pigs, research focussed mainly on the effects of smoke on airway hyperresponsiveness. Allergen-sensitized animals show an augmented bronchomotor response towards acute MS inhalation compared to non-sensitized animals, which is mediated by endogeneous tachykinins [115]. These neuropeptides affect airway smooth muscle tone, vascular permeability, mucus secretion and the release of inflammatory mediators, leading to neurogenic inflammation. Chronic MS exposure significantly increased airway hyperresponsiveness upon allergen challenge in sensitized animals, and upon capsaicin challenge independent of sensitization, indicating that MS can act as an adjuvant for both antigenic and neurogenic airway responsiveness [116].

The effects of **DEP **on sensitization and the development of allergic airway inflammation were evaluated in Brown Norway rats, using timothy grass pollen, house dust mite or OVA as allergen. As for mice, DEP exposure generally increased the levels of allergen-specific IgE and IgG [117-121]. Also increased eosinophilic airway inflammation [118,119,122] and hyperresponsiveness [118] could be demonstrated, albeit not in all models [117,120,121].

## Mechanistic view on the clinical impact of inhaled pollutants on asthma

**Figure 2:**
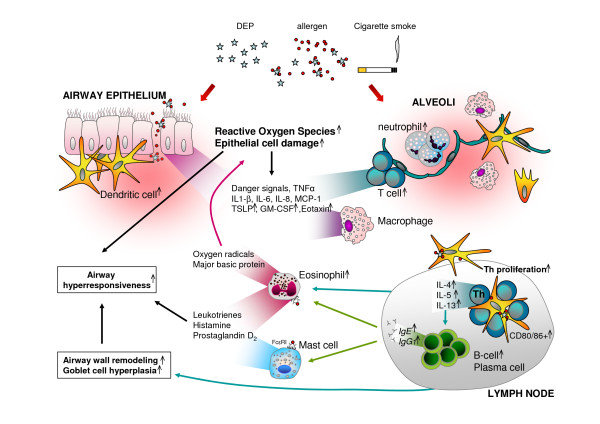
**Diesel exhaust particles (DEP) and cigarette smoke affect allergic sensitization and the development or exacerbation of asthma**. Similar proposed mechanisms for both inhalable pollutants, obtained from human, mouse and in vitro data are shown. Both DEP and cigarette smoke induce tissue damage and oxidative stress, resulting in a pulmonary inflammation with increased neutrophils and T-cells, and increased pro-inflammatory cytokines. This creates an environment which facilitates allergic sensitization. Cell types and mediators that are increased upon the combination of allergen and inhalable pollutant exposure (both DEP and cigarette smoke) and which are specifically associated with allergic asthma are indicated with a small black arrow.

The proposed mechanisms by which DEP and cigarette smoke favour the allergic sensitization, development and aggravation of asthma have been reviewed previously and are based on reports in mice and man [12,123,124]. Table 1 gives an overview of similarities and differences in mechanistic observations for DEP and cigarette smoke in both species. The effects of both inhalable pollutants show striking analogies (Figure 2). Inducing damage of the airway epithelium is probably the primary event for both DEP and cigarette smoke. This occurs through direct toxicity or oxidative stress, hereby inducing inflammatory cell recruitment and inflammatory mediator release. Reactive oxygen species (ROS), released directly or indirectly by mononuclear phagocytes, can contribute to airway inflammation through the induction of cytokines, chemokines and adhesion molecules via the NF-κB pathway and mitogen-activated protein kinase cascades in macrophages and epithelial cells. This inflamed pulmonary environment has the ability to attract dendritic cells and enhance their activation, thereby increasing allergen capture and transport to the lymph nodes [72,125]. Moreover, tobacco smoke and DEP induce epithelial release of the growth factors GM-CSF and thymic stromal lymphopoietin (TSLP), which can stimulate dendritic cell activation [126-129]. Oxidative stress can by itself also affect epithelial cell surface integrity. Co-administration of allergen and inhalable pollutant could thus facilitate penetration of allergen into the epithelial layer, resulting in a more efficient uptake and subsequent antigen presentation by dendritic cells. DEP can adsorb allergens onto their surface and act as carriers to increase allergen deposition into the respiratory tract [130]. This, as well as the decreased phagocytic capacity of alveolar macrophages, could prolong allergen exposure and increase immune reactivity.

**Table 1:** Mechanistic effects of tobacco smoke and diesel exhaust in human and mouse

TOBACCO SMOKE			DIESEL EXHAUST PARTICLES		
**Human**	**Mouse**	**BASAL EFFECTS**	**Human**	**Mouse**	

↑[100,144,145]	↑[100,146]	Oxidative Stress/Cell damage	↑* [15,147,148]	↑[15,86,149-151]	

↑[18,152]	↑[100,134,146,153,154], *f*[155]	TNFα	~[156,157]	↑[106,158-161], ↓[107]	

↑[18,152]	↑[100,162,163], *f *[162]	IL-1β	↑* [148], ~[156]	↑[135,150,161], ↓[159]	

↑[18,152]	↑[95,163], ~[153]	IL-6	↑[157,164], ~[165]	↑[158,160], *f*[161]	

↑[18,152]	↑[95,100,153,154,163]	IL-8/KC	↑[17,156,166], ↑* [148,167-170], ~[165]	↑[125,135,160]	

↑[18,152]	↑[95,100,134,153,154]	MCP-1	↑* [170]	↑[125,150,160], ~[111]	

↑,~[171]	↑ [95], *f*[171]	GM-CSF	↑* [148,167-169], ~[156,165], f* [128]	↑[172], *f*[172], ↓[106], ~[104]	

~[173]	~[89]	Eotaxin	↑* [174], ~[175]	↑[112], ~[111]	

↑ [176]	↑[126]	TSLP	↑* [127], f* [127]	▪	

↑ [177-179]	↑[134,154,180], ↓[181]	Dendritic cell number	▪	↑[125]	

▪	↑[72], ~[181]	DC transport of antigen	▪	↑[125]	

↑[177,182]	↑[72,134,180], ↓[181]	Costimulatory molecule expression	↑* [127,128]	↑[84,125]	

~or ↑[183,184]	▪	Eosinophil number or degranulation	↑* [185], ~[175,186],	↑[109], ~[106,107,110]	

↑[16,18,101,187]	↑[134,153,154,180]	Neutrophil number	↑[15,17,157,175,186,188]	↑[86,104,106,107,110,112,125,158,159]	

↑[16,101]	↑[134,153,154,180]	T or B-cell number	↑[15,17,157,175,188], ~[186]	↑[159], ~[104,110]	

↑[28,44], ~[25,27]	↑[95], ~[93]	IgE	↑[15,123,189]	~[86,104,106,107,109,110]	

↑[190]	~[153]	IgG	~[123,189]	~[86,104,106,107,109,110]	

**Human**	**Mouse**	**EFFECTS IN ALLERGIC DISEASE**	**Human**	**Mouse**	

▪	*f *[73], ↑[63,73], ~[71]	GM-CSF	↑* [168,169], ~[165]	↑[85], ↓[107]	

↑[173]	↑[70,89,93]	Eotaxin	▪	↑[85,88,111,112]	

↑[46]	↑[63,72,90], ↓[70,73]	Neutrophil number	↑[166], ~[17]	↑[104-106,108,110,112]	

↓[191]	↑[64,72,73,93,94,192], ↓[70,92]	T or B-lymphocyte number	↑[193], ~[17]	↑[84,85,104,105,108-111]	

↓[173,194]	↑[63-65,68,69,71-73,89,94], ↓[70,96], ~[92]	Eosinophil number	~[17]	↑[83,85,87,88,104-106,109-111]	

▪	↓[96]	T- or B-cell proliferation	↑[193]	↑[76,77,79,84,131]	

↓[191]	↑[71-73,93,94], ↓[70]	Dendritic cell number	▪	▪	

▪	↑[72]	Costimulatory molecule expression	↑[195]	↑[84,131]	

↑[194]	↑[90]	Mast cell number	↑[166]	↑[108]	

↑[29]	↑[64,70], ↓[96]	IL-4	↑[55,123,193]	↑[76-78,84,131], ↓ [107]	

↑[29], ↓[173]	↑[63,65,68,70,71,73,89], ↓[96]	IL-5	↑[85,123]	↑[85,88,104-107,109-112,131]	

↑[29]	↑[65,70,72]	IL-13	↑[123]	↑[112,131]	

↑[29,44]	↑[63-65,71,73,93], ~[67-70,90,92,94]	IgE	↑[15,55,56,123]	↑[74-84,86,87,104,105,107,108], ~[106,110]	

▪	↑[63-65,71,73], ↓[96]	IgG	↑[56,123], ~[55]	↑[81,82,84-86,88,104,105,107-109,111,112], ~[106,110]	

↑[29]	▪	Histamine	↑[15,196]	▪	

↑[30,32]	↑[66,68,68,69,89-91,93], ~[67,71], ↓[70,92]	Airway hyperresponsiveness	↑[15,17,59]	↑ [105-108]	

↑[194]	↑[89,90], ~[95]	Airway wall remodeling	▪	↑[110]	

↑[194]	↑[72,73,89,90], ↓[70]	Goblet cell hyperplasia	▪	↑[83,85,87,106-108,110,111]	

▪: not described; ↑: increased; ~: no effect; ↓: decreased; *: in vitro data; *f*: functional involvement

Besides the effects on the innate immune response, DEP or cigarette smoke can affect the adaptive responses towards allergens by enhancing costimulatory molecule expression and T-cell proliferation in the draining lymph nodes [71,72,125,131]. This results in an increased expression of Th2 cytokines, such as IL-4, IL-5 and IL-13. Besides IL-5, also GM-CSF, and eotaxin are increased upon exposure to both inhalable pollutants and allergen. These mediators affect the eosinophil, one of the most prominent cells in the inflammation of allergic asthma, and are responsible for its maturation, survival and attraction to sites of inflammation. IL-4, IL-5 and IL-13 contribute to goblet cell hyperplasia, airway wall remodelling and airway hyperresponsiveness [129]. IgE and IgG production by B-cells is elevated by DEP and cigarette smoke through the action of IL-4. Binding of IgE, crosslinked with allergen, induces eosinophil and mast cell degranulation. The subsequent release of major basic protein and oxygen radicals induces bronchial inflammation, whereas histamine and leukotrienes induce airway hyperresponsiveness, thus further enhancing the effects of both inhaled pollutants on the asthmatic response.

In addition to the above mentioned and established mechanisms, there are newly emerging hypotheses by which inhalable pollutant exposure could affect asthma pathogenesis which merit further investigation [124]. The release of Damage Associated Molecular Patterns (DAMPs) upon inhalable pollutant exposure could play an important role in the asthma pathogenesis. DAMPs are danger signals that can be actively or passively released upon tissue damage or cellular stress. Several of these molecules can stimulate DC maturation and thus act as endogenous adjuvants, such as high mobility group box 1 (HMGB1) protein, heat shock proteins (HSP), adenosine-triphosphate (ATP) and uric acid [124]. The induction of somatic mutations by oxidative DNA damage in lung epithelial barrier cells is also a danger signal that could lead to DC polarization [132]. The activation of the inflammasomes, major intracellular immune response systems that sense danger, is an interesting pathway for future research [133]. Toll-like receptor signalling, which is activated upon recognition of Pathogen Associated Molecular Patterns (PAMPs) such as LPS, microbial sugars or DNA or RNA is another possible mechanism. For TLR4 it has been demonstrated that it is involved in pulmonary inflammation induced by tobacco smoke and DEP as such [134,135]. In a model of allergic sensitization induced by tobacco smoke exposure, however, both TLR4 and MyD88 appeared not to be required [72]. Recently, a role for TLR9 in an ETS/OVA model was reported [136]. It is of great interest to elucidate the putative pathways further and to determine if and at what level tobacco smoke and DEP show discrepant effects in their ability to induce and aggravate allergic inflammation.

## Relevance of data from murine research: pro/con of mouse models

In general, the observed effects of ETS, MS and DEP on the development and aggravation of allergic inflammation in mice correspond well with the observations in asthma patients. The clinical findings for DEP are strongly supported by data obtained in mouse experimental models. In the case of ETS and MS, both clinical findings and mouse data indicate that the relationship between smoke exposure and allergic sensitization and development of clinical asthma is less convincing than for DEP.

Mouse models have several limitations which should be considered when the effects of inhaled pollutant exposure on allergic inflammation are investigated [4,137,138]: i) Mice do not spontaneously develop asthma. ii) The frequently used model-allergen OVA has little biological relevance. However, aggravating effects of pollutant exposure can also be demonstrated in house dust mite models or models without intraperitoneal injections. iii) There are considerable differences in human and mouse immunology [4,137,138]. Species differences in number and size of the alveolar macrophages, for example, can affect the efficiency of alveolar clearance. iiii) Differences in respiratory physiology and pharmacology can have implications in view of the effects of exposure to inhalational pollutants. Mice are obligate nose breathers, incapable of mouth breathing. The oral breathing in humans bypasses the effective air cleaning capacity of the nose. Mice have lower number of cilia, fewer Clara cells and restriction of submucosal glands to the trachea resulting in a different filtering of inhaled particles compared to man [101]. This can have an impact on the distribution of inhaled particles throughout the respiratory tract [139,140]. Mice furthermore do not have a cough reflex and many mediators such as histamine and tachykinins have different pharmacological effects in humans, which complicates mechanistic analysis of the effects of inhaled pollutants on bronchial hyperresponsiveness [101,138]. iiiii) Finally, also anatomical and developmental differences can be important. Differences in pulmonary lobulation and bronchial branching (six airway generations in mice versus 23 airway generations in humans), which are already present during the embryonic stage of lung development, can affect particle distribution [139]. In this view, the anatomical location of specific pathological mechanisms induced by particles, such as remodeling, might be different in mice compared to man. In contrast to humans, rodents also do not have respiratory bronchioles. This results in a faster alveolar clearance in rodents, since bronchioles impede rapid clearance of particles from the alveoli.

Species differences may become particularly important in studies on the effects of inhaled pollutant exposure on asthma development early in life. Lung development encompasses different phases of cellular differentiation, branching morphogenesis and overall lung growth, which each can be differently affected depending on the timing of inhaled pollutant exposure [141]. Although the rodent and human respiratory system go across identical phases of development, the timing of each phase is markedly different. In humans, lung growth is essentially complete by the end of adolescence, whereas mouse lungs are more fully developed at birth, implicating that effects of postnatal pollutant exposure in both species cannot be directly compared [142].

However, despite these concerns, rodent models are a valuable tool to test hypotheses which are generated by epidemiological research [4,138] and give more insights in how inhaled pollutant exposure can induce asthma development. i) Mouse models mimic important features of asthma, such as the pulmonary inflammation, remodelling and airway hyperresponsiveness, so the impact of inhaled pollutants on these features can be easily evaluated (Table 1). ii) Important analogies concerning the effects of *in utero *exposures are reported. For example, maternal smoking induces airway remodelling in mice offspring [90], which mimics increased lung remodelling due to *in utero *smoke exposure in children who died from sudden infant death syndrome [143]. Also effects of perinatal ETS exposure on the development of pulmonary function decrements in children can be well modelled in rats [141]. iii) Some important, but more general, advantages of using mouse asthma models are the relatively low cost, the availability of different inbred strains with different immunologic and physiologic properties, the numerous tools for experimental studies, the availability of the complete DNA sequence, and the existence of genetically modified strains [138]. Mice can thus be used for mechanistic studies that are not possible in humans due to ethical reasons. Such mechanistic studies, e.g. using genetically modified mice, are essential for elucidating the contribution of specific mediators or cell types (e.g. dendritic cells).

Mouse models can provide us with a biological basis for the observed associations between air pollution and allergic asthma in humans. They should of course mimic the clinical observations as closely as possible. Distinguishing between sensitization, development and aggravation and carefully selecting the appropriate models to answer specific research questions are therefore essential in studying the impact of inhalable pollutants on the pathogenesis of asthma.

## Conclusions

Exposure to inhalable pollutants is an important factor which affects sensitization, development and aggravation of asthma. Although clinical and epidemiological studies provide direct indications about the importance of inhaled pollutants in the pathogenesis of asthma, data from mice hold promise to provide mechanistic clues. We here reviewed the excess of mouse models that are available, focusing on unmet needs that would allow determining the critical mediators involved in the effects of the aforementioned pollutants on the different stages of the disease.

## Authors' contributions

TM and SP performed the literature search and collected all relevant publications. TM drafted the manuscript. TM and EL designed the figures. GJ, KT and DC assisted with the design of the review. All authors helped to draft the manuscript and critically read and approved the final manuscript.

## Competing interests

The authors declare that they have no competing interests.

## Supplementary Material

Additional file 1 **Table 2: Effects of environmental tobacco smoke (ETS) on murine allergic sensitization**. Table 2 provides a detailed overview of methodologies and results from murine models that examine the effects of ETS on allergic sensitization Click here for file

Additional file 2 **Table 3: Effects of mainstream cigarette smoke (MS) on murine allergic sensitization**. Table 3 provides a detailed overview of methodologies and results from murine models that examine the effects of MS on allergic sensitization Click here for file

Additional file 3 **Table 4: Effects of diesel exhaust particles (DEP) on murine allergic sensitization**. Table 4 provides a detailed overview of methodologies and results from murine models that examine the effects of DEP on allergic sensitization Click here for file

Additional file 4 **Table 5: Effects of environmental tobacco smoke (ETS) on development or aggravation of asthma in murine models**. Table 5 provides a detailed overview of methodologies and results from murine models that examine the effects of ETS on development or aggravation of asthma Click here for file

Additional file 5 **Table 6: Effects of mainstream cigarette smoke (MS) on development or aggravation of asthma in murine models**. Table 6 provides a detailed overview of methodologies and results from murine models that examine the effects of MS on development or aggravation of asthma Click here for file

Additional file 6 **Table 7: Effects of diesel exhaust particles (DEP) on development or aggravation of asthma in murine models**. Table 7 provides a detailed overview of methodologies and results from murine models that examine the effects of DEP on development or aggravation of asthma Click here for file

## References

[B1] Global Initiative for Asthma Global strategy for asthma management and prevention: updated 2006 2008 http://www.ginasthma.org

[B2] Eder W Ege MJ von Mutius E The asthma epidemic N Engl J Med 2006 355 2226 2235 10.1056/NEJMra054308 17124020

[B3] Umetsu DT McIntire JJ Akbari O Macaubas C DeKruyff RH Asthma: an epidemic of dysregulated immunity Nat Immunol 2002 3 715 720 10.1038/ni0802-715 12145657

[B4] Zosky GR Sly PD Animal models of asthma Clin Exp Allergy 2007 37 973 988 10.1111/j.1365-2222.2007.02740.x 17581191

[B5] World Health Organisation - IARC Tobacco Smoking and Tobacco Smoke, Summary of Data Reported and Evaluation Evaluation 2007 Chapter 5.5 83

[B6] Stedman RL The chemical composition of tobacco and tobacco smoke Chem Rev 1968 68 153 207 10.1021/cr60252a002 4868017

[B7] Swauger JE Steichen TJ Murphy PA Kinsler S An analysis of the mainstream smoke chemistry of samples of the U.S. cigarette market acquired between 1995 and 2000 Regul Toxicol Pharmacol 2002 35 142 156 10.1006/rtph.2001.1521 12052000

[B8] Thielen A Klus H Muller L Tobacco smoke: Unraveling a controversial subject Exp Toxicol Pathol 2008 60 2-3 141 156 10.1016/j.etp.2008.01.014 18485684

[B9] Hogg JC Chu F Utokaparch S Woods R Elliott WM Buzatu L Cherniack RM Rogers RM Sciurba FC Coxson HO Pare PD The nature of small-airway obstruction in chronic obstructive pulmonary disease N Engl J Med 2004 350 2645 2653 10.1056/NEJMoa032158 15215480

[B10] Aoshiba K Nagai A Differences in airway remodeling between asthma and chronic obstructive pulmonary disease Clin Rev Allergy Immunol 2004 27 35 43 10.1385/CRIAI:27:1:035 15347849

[B11] World Health Organisation Air quality guidelines for particulate matter, ozone, nitrogen dioxide and sulfur dioxide Global update Summary of risk assessment 2005 http://whqlibdoc.who.int/hq/2006/WHO_SDE_PHE_OEH_06.02_eng.pdf 34662007

[B12] Salvi S Holgate ST Mechanisms of particulate matter toxicity Clin Exp Allergy 1999 29 1187 1194 10.1046/j.1365-2222.1999.00576.x 10469026

[B13] Wichmann HE Diesel exhaust particles Inhal Toxicol 2007 19 Suppl 1 241 244 10.1080/08958370701498075 17886072

[B14] Sydbom A Blomberg A Parnia S Stenfors N Sandstrom T Dahlen SE Health effects of diesel exhaust emissions Eur Respir J 2001 17 733 746 10.1183/09031936.01.17407330 11401072

[B15] Riedl M Diaz-Sanchez D Biology of diesel exhaust effects on respiratory function J Allergy Clin Immunol 2005 115 221 228 10.1016/j.jaci.2004.11.047 15696072

[B16] Majo J Ghezzo H Cosio MG Lymphocyte population and apoptosis in the lungs of smokers and their relation to emphysema Eur Respir J 2001 17 946 953 10.1183/09031936.01.17509460 11488331

[B17] Stenfors N Nordenhall C Salvi SS Mudway I Soderberg M Blomberg A Helleday R Levin JO Holgate ST Kelly FJ Frew AJ Sandstrom T Different airway inflammatory responses in asthmatic and healthy humans exposed to diesel Eur Respir J 2004 23 82 86 10.1183/09031936.03.00004603 14738236

[B18] Kuschner WG D'Alessandro A Wong H Blanc PD Dose-dependent cigarette smoking-related inflammatory responses in healthy adults Eur Respir J 1996 9 1989 1994 10.1183/09031936.96.09101989 8902455

[B19] Hodge S Hodge G Ahern J Jersmann H Holmes M Reynolds PN Smoking alters alveolar macrophage recognition and phagocytic ability: implications in chronic obstructive pulmonary disease Am J Respir Cell Mol Biol 2007 37 748 755 10.1165/rcmb.2007-0025OC 17630319

[B20] Bousquet J Vignola AM Exposure to environmental tobacco smoke and adult asthma Allergy 2001 56 466 469 10.1034/j.1398-9995.2001.056006466.x 11421889

[B21] von Mutius E Environmental factors influencing the development and progression of pediatric asthma J Allergy Clin Immunol 2002 109 S525 S532 10.1067/mai.2002.124565 12063508

[B22] Skorge TD Eagan TM Eide GE Gulsvik A Bakke PS The adult incidence of asthma and respiratory symptoms by passive smoking in uterus or in childhood Am J Respir Crit Care Med 2005 172 61 66 10.1164/rccm.200409-1158OC 15805186

[B23] Cook DG Strachan DP Health effects of passive smoking. 3. Parental smoking and prevalence of respiratory symptoms and asthma in school age children Thorax 1997 52 1081 1094 10.1136/thx.52.12.1081 9516904 PMC1758471

[B24] Larsson ML Frisk M Hallstrom J Kiviloog J Lundback B Environmental tobacco smoke exposure during childhood is associated with increased prevalence of asthma in adults Chest 2001 120 711 717 10.1378/chest.120.3.711 11555497

[B25] Janson C Chinn S Jarvis D Zock JP Toren K Burney P Effect of passive smoking on respiratory symptoms, bronchial responsiveness, lung function, and total serum IgE in the European Community Respiratory Health Survey: a cross-sectional study Lancet 2001 358 2103 2109 10.1016/S0140-6736(01)07214-2 11784622

[B26] Larsson ML Loit HM Meren M Polluste J Magnusson A Larsson K Lundback B Passive smoking and respiratory symptoms in the FinEsS Study Eur Respir J 2003 21 672 676 10.1183/09031936.03.00033702 12762355

[B27] Strachan DP Cook DG Health effects of passive smoking. 5. Parental smoking and allergic sensitisation in children Thorax 1998 53 117 123 10.1136/thx.53.2.117 9624297 PMC1758719

[B28] Lannero E Wickman M van Hage M Bergstrom A Pershagen G Nordvall L Exposure to environmental tobacco smoke and sensitisation in children Thorax 2008 63 172 176 10.1136/thx.2007.079053 18089631

[B29] Diaz-Sanchez D Rumold R Gong H Jr Challenge with environmental tobacco smoke exacerbates allergic airway disease in human beings J Allergy Clin Immunol 2006 118 441 446 10.1016/j.jaci.2006.04.047 16890770

[B30] Coultas DB Health effects of passive smoking. 8. Passive smoking and risk of adult asthma and COPD: an update Thorax 1998 53 381 387 10.1136/thx.53.5.381 9708231 PMC1745215

[B31] Eisner MD Yelin EH Katz PP Earnest G Blanc PD Exposure to indoor combustion and adult asthma outcomes: environmental tobacco smoke, gas stoves, and woodsmoke Thorax 2002 57 973 978 10.1136/thorax.57.11.973 12403881 PMC1746223

[B32] Joad JP Smoking and pediatric respiratory health Clin Chest Med 2000 21 37 46 vii-viii. 10.1016/S0272-5231(05)70006-X 10763088

[B33] Mannino DM Homa DM Redd SC Involuntary smoking and asthma severity in children: data from the Third National Health and Nutrition Examination Survey Chest 2002 122 409 415 10.1378/chest.122.2.409 12171810

[B34] Tatum AJ Shapiro GG The effects of outdoor air pollution and tobacco smoke on asthma Immunol Allergy Clin North Am 2005 25 15 30 10.1016/j.iac.2004.09.003 15579362

[B35] Siroux V Pin I Oryszczyn MP Le Moual N Kauffmann F Relationships of active smoking to asthma and asthma severity in the EGEA study. Epidemiological study on the Genetics and Environment of Asthma Eur Respir J 2000 15 470 477 10.1034/j.1399-3003.2000.15.08.x 10759439

[B36] Piipari R Jaakkola JJ Jaakkola N Jaakkola MS Smoking and asthma in adults Eur Respir J 2004 24 734 739 10.1183/09031936.04.00116903 15516665

[B37] Lundback B Ronmark E Jonsson E Larsson K Sandstrom T Incidence of physician-diagnosed asthma in adults--a real incidence or a result of increased awareness? Report from The Obstructive Lung Disease in Northern Sweden Studies Respir Med 2001 95 685 692 10.1053/rmed.2001.1126 11530958

[B38] Gilliland FD Islam T Berhane K Gauderman WJ McConnell R Avol E Peters JM Regular Smoking and Asthma Incidence in Adolescents Am J Respir Crit Care Med 2006 174 1094 100 10.1164/rccm.200605-722OC 16973983 PMC2648110

[B39] Genuneit J Weinmayr G Radon K Dressel H Windstetter D Rzehak P Vogelberg C Leupold W Nowak D von Mutius E Weiland SK Smoking and the incidence of asthma during adolescence: results of a large cohort study in Germany Thorax 2006 61 572 578 10.1136/thx.2005.051227 16537668 PMC2104663

[B40] Avila L Soto-Martinez ME Soto-Quiros ME Celedon JC Asthma, current wheezing, and tobacco use among adolescents and young adults in Costa Rica J Asthma 2005 42 543 547 10.1080/02770900500214791 16169786

[B41] Kim YK Kim SH Tak YJ Jee YK Lee BJ Kim SH Park HW Jung JW Bahn JW Chang YS Choi DC Chang SI Min KU Kim YY Cho SH High prevalence of current asthma and active smoking effect among the elderly Clin Exp Allergy 2002 32 1706 1712 10.1046/j.1365-2222.2002.01524.x 12653160

[B42] Annesi-Maesano I Oryszczyn MP Raherison C Kopferschmitt C Pauli G Taytard A Tunon de Lara M Vervloet D Charpin D Increased prevalence of asthma and allied diseases among active adolescent tobacco smokers after controlling for passive smoking exposure. A cause for concern? Clin Exp Allergy 2004 34 1017 1023 10.1111/j.1365-2222.2004.02002.x 15248844

[B43] Miyake Y Miyamoto S Ohya Y Sasaki S Matsunaga I Yoshida T Hirota Y Oda H Association of active and passive smoking with allergic disorders in pregnant Japanese women: baseline data from the Osaka Maternal and Child Health Study Ann Allergy Asthma Immunol 2005 94 644 651 15984596 10.1016/S1081-1206(10)61322-1

[B44] Oryszczyn MP Annesi-Maesano I Charpin D Paty E Maccario J Kauffmann F Relationships of active and passive smoking to total IgE in adults of the Epidemiological Study of the Genetics and Environment of Asthma, Bronchial Hyperresponsiveness, and Atopy (EGEA) Am J Respir Crit Care Med 2000 161 1241 1246 10764318 10.1164/ajrccm.161.4.9905027

[B45] Chaudhuri R McSharry C McCoard A Livingston E Hothersall E Spears M Lafferty J Thomson NC Role of symptoms and lung function in determining asthma control in smokers with asthma Allergy 2008 63 132 135 18053022 10.1111/j.1398-9995.2007.01538.x

[B46] Boulet LP Lemiere C Archambault F Carrier G Descary MC Deschesnes F Smoking and asthma: clinical and radiologic features, lung function, and airway inflammation Chest 2006 129 661 668 10.1378/chest.129.3.661 16537865

[B47] James AL Palmer LJ Kicic E Maxwell PS Lagan SE Ryan GF Musk AW Decline in lung function in the Busselton Health Study: the effects of asthma and cigarette smoking Am J Respir Crit Care Med 2004 171 109 114 10.1164/rccm.200402-230OC 15486340

[B48] Beeh KM Micke P Ksoll M Buhl R Cigarette smoking, but not sensitization to Alternaria, is associated with severe asthma in urban patients J Asthma 2001 38 41 49 10.1081/JAS-100000020 11256553

[B49] Thomson NC Spears M The influence of smoking on the treatment response in patients with asthma Curr Opin Allergy Clin Immunol 2005 5 57 63 15643345 10.1097/00130832-200502000-00011

[B50] Braback L Forsberg B Does traffic exhaust contribute to the development of asthma and allergic sensitization in children: findings from recent cohort studies Environ Health 2009 8 17 10.1186/1476-069X-8-17 19371435 PMC2674435

[B51] Heinrich J Wichmann HE Traffic related pollutants in Europe and their effect on allergic disease Curr Opin Allergy Clin Immunol 2004 4 341 348 10.1097/00130832-200410000-00003 15349031

[B52] Polosa R Salvi S Di Maria GU Allergic susceptibility associated with diesel exhaust particle exposure: clear as mud Arch Environ Health 2002 57 188 193 10.1080/00039890209602935 12507171

[B53] Morgenstern V Zutavern A Cyrys J Brockow I Koletzko S Kramer U Behrendt H Herbarth O von Berg A Bauer CP Wichmann HE Heinrich J Atopic diseases, allergic sensitization, and exposure to traffic-related air pollution in children Am J Respir Crit Care Med 2008 177 1331 1337 10.1164/rccm.200701-036OC 18337595

[B54] Nordling E Berglind N Melen E Emenius G Hallberg J Nyberg F Pershagen G Svartengren M Wickman M Bellander T Traffic-related air pollution and childhood respiratory symptoms, function and allergies Epidemiology 2008 19 401 408 10.1097/EDE.0b013e31816a1ce3 18379426

[B55] Diaz-Sanchez D Garcia MP Wang M Jyrala M Saxon A Nasal challenge with diesel exhaust particles can induce sensitization to a neoallergen in the human mucosa J Allergy Clin Immunol 1999 104 1183 1188 10.1016/S0091-6749(99)70011-4 10588999

[B56] Diaz-Sanchez D Tsien A Fleming J Saxon A Combined diesel exhaust particulate and ragweed allergen challenge markedly enhances human in vivo nasal ragweed-specific IgE and skews cytokine production to a T helper cell 2-type pattern J Immunol 1997 158 2406 2413 9036991

[B57] Boezen M Schouten J Rijcken B Vonk J Gerritsen J Zee S van der Hoek G Brunekreef B Postma D Peak expiratory flow variability, bronchial responsiveness, and susceptibility to ambient air pollution in adults Am J Respir Crit Care Med 1998 158 1848 1854 9847277 10.1164/ajrccm.158.6.9804072

[B58] Holguin F Traffic, outdoor air pollution, and asthma Immunol Allergy Clin North Am 2008 28 577 588 10.1016/j.iac.2008.03.008 18572108

[B59] Nordenhall C Pourazar J Ledin MC Levin JO Sandstrom T Adelroth E Diesel exhaust enhances airway responsiveness in asthmatic subjects Eur Respir J 2001 17 909 915 10.1183/09031936.01.17509090 11488325

[B60] McCreanor J Cullinan P Nieuwenhuijsen MJ Stewart-Evans J Malliarou E Jarup L Harrington R Svartengren M Han IK Ohman-Strickland P Chung KF Zhang J Respiratory effects of exposure to diesel traffic in persons with asthma N Engl J Med 2007 357 2348 2358 10.1056/NEJMoa071535 18057337

[B61] Holt PG Batty JE Turner KJ Inhibition of specific IgE responses in mice by pre-exposure to inhaled antigen Immunology 1981 42 409 417 7203528 PMC1458442

[B62] Seymour BW Gershwin LJ Coffman RL Aerosol-induced immunoglobulin (Ig)-E unresponsiveness to ovalbumin does not require CD8+ or T cell receptor (TCR)-gamma/delta+ T cells or interferon (IFN)-gamma in a murine model of allergen sensitization J Exp Med 1998 187 721 731 10.1084/jem.187.5.721 9480982 PMC2212168

[B63] Rumold R Jyrala M Diaz-Sanchez D Secondhand smoke induces allergic sensitization in mice J Immunol 2001 167 4765 4770 11591808 10.4049/jimmunol.167.8.4765

[B64] Seymour BW Pinkerton KE Friebertshauser KE Coffman RL Gershwin LJ Second-hand smoke is an adjuvant for T helper-2 responses in a murine model of allergy J Immunol 1997 159 6169 6175 9550419

[B65] Seymour BW Friebertshauser KE Peake JL Pinkerton KE Coffman RL Gershwin LJ Gender differences in the allergic response of mice neonatally exposed to environmental tobacco smoke Dev Immunol 2002 9 47 54 10.1080/1044667021000003989 12353662 PMC2276086

[B66] Barrett EG Wilder JA March TH Espindola T Bice DE Cigarette smoke-induced airway hyperresponsiveness is not dependent on elevated immunoglobulin and eosinophilic inflammation in a mouse model of allergic airway disease Am J Respir Crit Care Med 2002 165 1410 1418 10.1164/rccm.2106029 12016105

[B67] Bowles K Horohov D Paulsen D Leblanc C Littlefield-Chabaud M Ahlert T Ahlert K Pourciau S Penn A Exposure of adult mice to environmental tobacco smoke fails to enhance the immune response to inhaled antigen Inhal Toxicol 2005 17 43 51 10.1080/08958370590885690 15764482

[B68] Penn AL Rouse RL Horohov DW Kearney MT Paulsen DB Lomax L In utero exposure to environmental tobacco smoke potentiates adult responses to allergen in BALB/c mice Environ Health Perspect 2007 115 548 555 17450223 10.1289/ehp.9780 PMC1852677

[B69] Seymour BW Schelegle ES Pinkerton KE Friebertshauser KE Peake JL Kurupd VP Coffman RL Gershwin LJ Second-hand smoke increases bronchial hyperreactivity and eosinophilia in a murine model of allergic aspergillosis Clin Dev Immunol 2003 10 35 42 10.1080/10446670310001598483 14575156 PMC2270675

[B70] Robbins CS Pouladi MA Fattouh R Dawe DE Vujicic N Richards CD Jordana M Inman MD Stampfli MR Mainstream cigarette smoke exposure attenuates airway immune inflammatory responses to surrogate and common environmental allergens in mice, despite evidence of increased systemic sensitization J Immunol 2005 175 2834 2842 16116169 10.4049/jimmunol.175.5.2834

[B71] Moerloose KB Robays LJ Maes T Brusselle GG Tournoy KG Joos GF Cigarette smoke exposure facilitates allergic sensitization in mice Respir Res 2006 7 49 10.1186/1465-9921-7-49 16571114 PMC1458334

[B72] Robays LJ Lanckacker EA Moerloose KB Maes T Bracke KR Brusselle GG Joos GF Vermaelen KY Concomitant inhalation of cigarette smoke and aerosolized protein activates airway dendritic cells and induces allergic airway inflammation in a TLR-independent way J Immunol 2009 183 2758 2766 10.4049/jimmunol.0802204 19635922

[B73] Trimble NJ Botelho FM Bauer CM Fattouh R Stampfli MR Adjuvant and anti-inflammatory properties of cigarette smoke in murine allergic airway inflammation Am J Respir Cell Mol Biol 2009 40 38 46 10.1165/rcmb.2008-0107OC 18635815

[B74] Muranaka M Suzuki S Koizumi K Takafuji S Miyamoto T Ikemori R Tokiwa H Adjuvant activity of diesel-exhaust particulates for the production of IgE antibody in mice J Allergy Clin Immunol 1986 77 616 623 10.1016/0091-6749(86)90355-6 2420853

[B75] Takafuji S Suzuki S Koizumi K Tadokoro K Miyamoto T Ikemori R Muranaka M Diesel-exhaust particulates inoculated by the intranasal route have an adjuvant activity for IgE production in mice J Allergy Clin Immunol 1987 79 639 645 10.1016/S0091-6749(87)80161-6 2435776

[B76] Fujimaki H Nohara O Ichinose T Watanabe N Saito S IL-4 production in mediastinal lymph node cells in mice intratracheally instilled with diesel exhaust particulates and antigen Toxicology 1994 92 261 268 10.1016/0300-483X(94)90182-1 7524198

[B77] Fujimaki H Saneyoshi K Nohara O Shiraishi F Imai T Intranasal instillation of diesel exhaust particulates and antigen in mice modulated cytokine productions in cervical lymph node cells Int Arch Allergy Immunol 1995 108 268 273 10.1159/000237163 7580292

[B78] Fujimaki H Saneyoshi K Shiraishi F Imai T Endo T Inhalation of diesel exhaust enhances antigen-specific IgE antibody production in mice Toxicology 1997 116 227 233 10.1016/S0300-483X(96)03539-1 9020525

[B79] Lovik M Hogseth AK Gaarder PI Hagemann R Eide I Diesel exhaust particles and carbon black have adjuvant activity on the local lymph node response and systemic IgE production to ovalbumin Toxicology 1997 121 165 178 10.1016/S0300-483X(97)00075-9 9230448

[B80] Nilsen A Hagemann R Eide I The adjuvant activity of diesel exhaust particles and carbon black on systemic IgE production to ovalbumin in mice after intranasal instillation Toxicology 1997 124 225 232 10.1016/S0300-483X(97)00150-9 9482124

[B81] Heo Y Saxon A Hankinson O Effect of diesel exhaust particles and their components on the allergen-specific IgE and IgG1 response in mice Toxicology 2001 159 143 158 10.1016/S0300-483X(00)00418-2 11223170

[B82] Suzuki T Kanoh T Ishimori M Ikeda S Ohkuni H Adjuvant activity of diesel exhaust particulates (DEP) in production of anti-IgE and anti-IgG1 antibodies to mite allergen in mice J Clin Lab Immunol 1996 48 187 199 9394242

[B83] Ichinose T Takano H Miyabara Y Yanagisawa R Sagai M Murine strain differences in allergic airway inflammation and immunoglobulin production by a combination of antigen and diesel exhaust particles Toxicology 1997 122 183 192 10.1016/S0300-483X(97)00096-6 9328218

[B84] van Zijverden M Pijl A van der Bol M van Pinxteren FA de Haar C Penninks AH Van Loveren H Pieters R Diesel exhaust, carbon black, and silica particles display distinct Th1/Th2 modulating activity Toxicol Appl Pharmacol 2000 168 131 139 10.1006/taap.2000.9013 11032768

[B85] Sadakane K Ichinose T Takano H Yanagisawa R Sagai M Yoshikawa T Shibamoto T Murine strain differences in airway inflammation induced by diesel exhaust particles and house dust mite allergen Int Arch Allergy Immunol 2002 128 220 228 10.1159/000064255 12119504

[B86] Whitekus MJ Li N Zhang M Wang M Horwitz MA Nelson SK Horwitz LD Brechun N Diaz-Sanchez D Nel AE Thiol antioxidants inhibit the adjuvant effects of aerosolized diesel exhaust particles in a murine model for ovalbumin sensitization J Immunol 2002 168 2560 2567 11859152 10.4049/jimmunol.168.5.2560

[B87] Steerenberg PA van Dalen WJ Withagen CE Dormans JA Van Loveren H Optimization of route of administration for coexposure to ovalbumin and particle matter to induce adjuvant activity in respiratory allergy in the mouse Inhal Toxicol 2003 15 1309 1325 10.1080/08958370390241786 14569495

[B88] Ichinose T Takano H Sadakane K Yanagisawa R Yoshikawa T Sagai M Shibamoto T Mouse strain differences in eosinophilic airway inflammation caused by intratracheal instillation of mite allergen and diesel exhaust particles J Appl Toxicol 2004 24 69 76 10.1002/jat.949 14745849

[B89] Min MG Song DJ Miller M Cho JY McElwain S Ferguson P Broide DH Coexposure to environmental tobacco smoke increases levels of allergen-induced airway remodeling in mice J Immunol 2007 178 5321 5328 17404317 10.4049/jimmunol.178.8.5321

[B90] Blacquiere MJ Timens W Melgert BN Geerlings M Postma DS Hylkema MN Maternal smoking during pregnancy induces airway remodelling in mice offspring Eur Respir J 2009 33 1133 1140 10.1183/09031936.00129608 19129273

[B91] Singh SP Barrett EG Kalra R Razani-Boroujerdi S Langley RJ Kurup V Tesfaigzi Y Sopori ML Prenatal cigarette smoke decreases lung cAMP and increases airway hyperresponsiveness Am J Respir Crit Care Med 2003 168 342 347 10.1164/rccm.200211-1262OC 12791581

[B92] Melgert BN Postma DS Geerlings M Luinge MA Klok PA Strate BW Van Der Kerstjens HA Timens W Hylkema MN Short-term smoke exposure attenuates ovalbumin-induced airway inflammation in allergic mice Am J Respir Cell Mol Biol 2004 30 880 885 10.1165/rcmb.2003-0178OC 14722223

[B93] Moerloose KB Pauwels RA Joos GF Short-term cigarette smoke exposure enhances allergic airway inflammation in mice Am J Respir Crit Care Med 2005 172 168 172 10.1164/rccm.200409-1174OC 15831841

[B94] Van Hove CL Moerloose K Maes T Joos GF Tournoy KG Cigarette smoke enhances Th-2 driven airway inflammation and delays inhalational tolerance Respir Res 2008 9 42 10.1186/1465-9921-9-42 18489797 PMC2408577

[B95] Melgert BN Timens W Kerstjens HA Geerlings M Luinge MA Schouten JP Postma DS Hylkema MN Effects of 4 months of smoking in mice with ovalbumin-induced airway inflammation Clin Exp Allergy 2007 37 1798 1808 10.1111/j.1365-2222.2007.02843.x 17941917

[B96] Thatcher TH Benson RP Phipps RP Sime PJ High dose but not low dose mainstream cigarette smoke suppresses allergic airway inflammation by inhibiting T cell function Am J Physiol Lung Cell Mol Physiol 2008 295 L412 21 10.1152/ajplung.00392.2007 18567739 PMC2536795

[B97] Chapman JT Otterbein LE Elias JA Choi AM Carbon monoxide attenuates aeroallergen-induced inflammation in mice Am J Physiol Lung Cell Mol Physiol 2001 281 L209 L216 11404264 10.1152/ajplung.2001.281.1.L209

[B98] Stampfli MR Anderson GP How cigarette smoke skews immune responses to promote infection, lung disease and cancer Nat Rev Immunol 2009 9 377 384 10.1038/nri2530 19330016

[B99] Brusselle GG Bracke KR Maes T D'hulst AI Moerloose KB Joos GF Pauwels RA Murine models of COPD Pulm Pharmacol Ther 2005 19 155 165 10.1016/j.pupt.2005.06.001 16084119

[B100] Churg A Cosio M Wright JL Mechanisms of cigarette smoke-induced COPD: insights from animal models Am J Physiol Lung Cell Mol Physiol 2008 294 L612 L631 10.1152/ajplung.00390.2007 18223159

[B101] Groneberg DA Chung KF Models of chronic obstructive pulmonary disease Respir Res 2004 5 18 10.1186/1465-9921-5-18 15522115 PMC533858

[B102] Bracke KR D'hulst AI Maes T Moerloose KB Demedts IK Lebecque S Joos GF Brusselle GG Cigarette Smoke-Induced Pulmonary Inflammation and Emphysema Are Attenuated in CCR6-Deficient Mice J Immunol 2006 177 4350 4359 16982869 10.4049/jimmunol.177.7.4350

[B103] Demoor T Bracke KR Maes T Vandooren B Elewaut D Pilette C Joos GF Brusselle GG Role of lymphotoxin-alpha in cigarette smoke-induced inflammation and lymphoid neogenesis Eur Respir J 2009 34 405 416 10.1183/09031936.00101408 19164352

[B104] Takano H Yoshikawa T Ichinose T Miyabara Y Imaoka K Sagai M Diesel exhaust particles enhance antigen-induced airway inflammation and local cytokine expression in mice Am J Respir Crit Care Med 1997 156 36 42 9230723 10.1164/ajrccm.156.1.9610054

[B105] Takano H Ichinose T Miyabara Y Yoshikawa T Sagai M Diesel exhaust particles enhance airway responsiveness following allergen exposure in mice Immunopharmacol Immunotoxicol 1998 20 329 336 10.3109/08923979809038548 9653676

[B106] Miyabara Y Ichinose T Takano H Lim HB Sagai M Effects of diesel exhaust on allergic airway inflammation in mice J Allergy Clin Immunol 1998 102 805 812 10.1016/S0091-6749(98)70021-1 9819298

[B107] Miyabara Y Takano H Ichinose T Lim HB Sagai M Diesel exhaust enhances allergic airway inflammation and hyperresponsiveness in mice Am J Respir Crit Care Med 1998 157 1138 1144 9563731 10.1164/ajrccm.157.4.9708066

[B108] Miyabara Y Ichinose T Takano H Sagai M Diesel exhaust inhalation enhances airway hyperresponsiveness in mice Int Arch Allergy Immunol 1998 116 124 131 10.1159/000023935 9652305

[B109] Miyabara Y Yanagisawa R Shimojo N Takano H Lim HB Ichinose T Sagai M Murine strain differences in airway inflammation caused by diesel exhaust particles Eur Respir J 1998 11 291 298 10.1183/09031936.98.11020291 9551727

[B110] Ichinose T Takano H Miyabara Y Sagai M Long-term exposure to diesel exhaust enhances antigen-induced eosinophilic inflammation and epithelial damage in the murine airway Toxicol Sci 1998 44 70 79 10.1093/toxsci/44.1.70 9720143

[B111] Ichinose T Takano H Sadakane K Yanagisawa R Kawazato H Sagai M Shibamoto T Differences in airway-inflammation development by house dust mite and diesel exhaust inhalation among mouse strains Toxicol Appl Pharmacol 2003 187 29 37 10.1016/S0041-008X(02)00038-8 12628582

[B112] Yanagisawa R Takano H Inoue KI Ichinose T Sadakane K Yoshino S Yamaki K Yoshikawa T Hayakawa K Components of diesel exhaust particles differentially affect Th1/Th2 response in a murine model of allergic airway inflammation Clin Exp Allergy 2006 36 386 395 10.1111/j.1365-2222.2006.02452.x 16499651

[B113] Matsumoto A Hiramatsu K Li Y Azuma A Kudoh S Takizawa H Sugawara I Repeated exposure to low-dose diesel exhaust after allergen challenge exaggerates asthmatic responses in mice Clin Immunol 2006 121 227 235 10.1016/j.clim.2006.08.003 16979384

[B114] Hao M Comier S Wang M Lee JJ Nel A Diesel exhaust particles exert acute effects on airway inflammation and function in murine allergen provocation models J Allergy Clin Immunol 2003 112 905 914 10.1016/j.jaci.2003.07.005 14610479

[B115] Wu ZX Zhou D Chen G Lee LY Airway hyperresponsiveness to cigarette smoke in ovalbumin-sensitized guinea pigs Am J Respir Crit Care Med 2000 161 73 80 10619800 10.1164/ajrccm.161.1.9809121

[B116] Bergren DR Tobacco smoke is an adjuvant for maintained airway sensitization in guinea pigs J Asthma 2007 44 723 728 10.1080/02770900701595642 17994401

[B117] Steerenberg PA Dormans JA van Doorn CC Middendorp S Vos JG Van Loveren H A pollen model in the rat for testing adjuvant activity of air pollution components Inhal Toxicol 1999 11 1109 1122 10.1080/089583799196619 10562699

[B118] Dong CC Yin XJ Ma JY Millecchia L Wu ZX Barger MW Roberts JR Antonini JM Dey RD Ma JK Effect of diesel exhaust particles on allergic reactions and airway responsiveness in ovalbumin-sensitized brown Norway rats Toxicol Sci 2005 88 202 212 10.1093/toxsci/kfi280 16107553

[B119] Al-Humadi NH Siegel PD Lewis DM Barger MW Ma JY Weissman DN Ma JK The effect of diesel exhaust particles (DEP) and carbon black (CB) on thiol changes in pulmonary ovalbumin allergic sensitized Brown Norway rats Exp Lung Res 2002 28 333 349 10.1080/01902140290091976 12097228

[B120] Dong CC Yin XJ Ma JY Millecchia L Barger MW Roberts JR Zhang XD Antonini JM Ma JK Exposure of brown Norway rats to diesel exhaust particles prior to ovalbumin (OVA) sensitization elicits IgE adjuvant activity but attenuates OVA-induced airway inflammation Toxicol Sci 2005 88 150 160 10.1093/toxsci/kfi298 16120749

[B121] Steerenberg PA Withagen CE Dormans JA van Dalen WJ Van Loveren H Casee FR Adjuvant activity of various diesel exhaust and ambient particles in two allergic models J Toxicol Environ Health A 2003 66 1421 1439 10.1080/15287390306415 12857633

[B122] Singh P Madden M Gilmour MI Effects of diesel exhaust particles and carbon black on induction of dust mite allergy in brown norway rats J Immunotoxicol 2005 2 41 49 10.1080/15476910590952458 18958658

[B123] Pandya RJ Solomon G Kinner A Balmes JR Diesel exhaust and asthma: hypotheses and molecular mechanisms of action Environ Health Perspect 2002 110 Suppl 1 103 112 11834468 10.1289/ehp.02110s1103 PMC1241152

[B124] Robays LJ Maes T Joos GF Vermaelen KY Between a cough and a wheeze: dendritic cells at the nexus of tobacco smoke-induced allergic airway sensitization Mucosal Immunol 2009 2 206 219 10.1038/mi.2009.7 19262504

[B125] Provoost S Maes T Willart MA Joos GF Lambrecht BN Tournoy KG Diesel exhaust particles stimulate adaptive immunity by acting on pulmonary dendritic cells J Immunol 2010 184 1 426 432 10.4049/jimmunol.0902564 19949085

[B126] Nakamura Y Miyata M Ohba T Ando T Hatsushika K Suenaga F Shimokawa N Ohnuma Y Katoh R Ogawa H Nakao A Cigarette smoke extract induces thymic stromal lymphopoietin expression, leading to T(H)2-type immune responses and airway inflammation J Allergy Clin Immunol 2008 122 1208 1214 10.1016/j.jaci.2008.09.022 18926564

[B127] Bleck B Tse DB Curotto de Lafaille MA Zhang F Reibman J Diesel exhaust particle-exposed human bronchial epithelial cells induce dendritic cell maturation and polarization via thymic stromal lymphopoietin J Clin Immunol 2008 28 147 156 10.1007/s10875-007-9149-0 18049884 PMC2757761

[B128] Bleck B Tse DB Jaspers I Curotto de Lafaille MA Reibman J Diesel exhaust particle-exposed human bronchial epithelial cells induce dendritic cell maturation J Immunol 2006 176 7431 7437 16751388 10.4049/jimmunol.176.12.7431

[B129] Hammad H Lambrecht BN Dendritic cells and epithelial cells: linking innate and adaptive immunity in asthma Nat Rev Immunol 2008 8 193 204 10.1038/nri2275 18301423

[B130] Knox RB Suphioglu C Taylor P Desai R Watson HC Peng JL Bursill LA Major grass pollen allergen Lol p 1 binds to diesel exhaust particles: implications for asthma and air pollution Clin Exp Allergy 1997 27 246 251 10.1111/j.1365-2222.1997.tb00702.x 9088650

[B131] Inoue K Koike E Takano H Yanagisawa R Ichinose T Yoshikawa T Effects of diesel exhaust particles on antigen-presenting cells and antigen-specific Th immunity in mice Exp Biol Med (Maywood) 2009 234 200 209 10.3181/0809-RM-285 19064938

[B132] Tzortzaki EG Siafakas NM A hypothesis for the initiation of COPD Eur Respir J 2009 34 310 315 10.1183/09031936.00067008 19648516

[B133] Martinon F Mayor A Tschopp J The inflammasomes: guardians of the body Annu Rev Immunol 2009 27 229 265 10.1146/annurev.immunol.021908.132715 19302040

[B134] Maes T Bracke KR Vermaelen KY Demedts IK Joos GF Pauwels RA Brusselle GG Murine TLR4 is implicated in cigarette smoke-induced pulmonary inflammation Int Arch Allergy Immunol 2006 141 354 368 10.1159/000095462 16940747

[B135] Inoue K Takano H Yanagisawa R Hirano S Ichinose T Shimada A Yoshikawa T The role of toll-like receptor 4 in airway inflammation induced by diesel exhaust particles Arch Toxicol 2006 80 275 279 10.1007/s00204-005-0040-6 16254717

[B136] Song DJ Min MG Miller M Cho JY Yum HY Broide DH Toll-Like Receptor-9 Agonist Inhibits Airway Inflammation, Remodeling and Hyperreactivity in Mice Exposed to Chronic Environmental Tobacco Smoke and Allergen Int Arch Allergy Immunol 2009 151 285 296 10.1159/000250437 19851071 PMC2853586

[B137] Mestas J Hughes CC Of mice and not men: differences between mouse and human immunology J Immunol 2004 172 2731 2738 14978070 10.4049/jimmunol.172.5.2731

[B138] Finkelman FD Wills-Karp M Usefulness and optimization of mouse models of allergic airway disease J Allergy Clin Immunol 2008 121 603 606 10.1016/j.jaci.2008.01.008 18328889 PMC3848028

[B139] Phalen RF Oldham MJ Wolff RK The relevance of animal models for aerosol studies J Aerosol Med Pulm Drug Deliv 2008 21 113 124 10.1089/jamp.2007.0673 18518837

[B140] Lippmann M Schlesinger RB Interspecies comparisons of particle deposition and mucociliary clearance in tracheobronchial airways J Toxicol Environ Health 1984 13 441 469 10.1080/15287398409530509 6376822

[B141] Pinkerton KE Joad JP Influence of air pollution on respiratory health during perinatal development Clin Exp Pharmacol Physiol 2006 33 269 272 10.1111/j.1440-1681.2006.04357.x 16487273

[B142] Wenzel S Holgate ST The mouse trap: It still yields few answers in asthma Am J Respir Crit Care Med 2006 174 1173 1176 10.1164/rccm.2609002 17110654

[B143] Elliot J Vullermin P Robinson P Maternal cigarette smoking is associated with increased inner airway wall thickness in children who die from sudden infant death syndrome Am J Respir Crit Care Med 1998 158 802 806 9731008 10.1164/ajrccm.158.3.9709055

[B144] Montuschi P Collins JV Ciabattoni G Lazzeri N Corradi M Kharitonov SA Barnes PJ Exhaled 8-isoprostane as an in vivo biomarker of lung oxidative stress in patients with COPD and healthy smokers Am J Respir Crit Care Med 2000 162 1175 1177 10988150 10.1164/ajrccm.162.3.2001063

[B145] Petecchia L Sabatini F Varesio L Camoirano A Usai C Pezzolo A Rossi GA Bronchial airway epithelial cell damage following exposure to cigarette smoke includes disassembly of tight junction components mediated by the extracellular signal-regulated kinase 1/2 pathway Chest 2009 135 1502 1512 10.1378/chest.08-1780 19447922

[B146] Sato A Hoshino Y Hara T Muro S Nakamura H Mishima M Yodoi J Thioredoxin-1 ameliorates cigarette smoke-induced lung inflammation and emphysema in mice J Pharmacol Exp Ther 2008 325 380 388 10.1124/jpet.107.134007 18256171

[B147] Li N Wang M Oberley TD Sempf JM Nel AE Comparison of the pro-oxidative and proinflammatory effects of organic diesel exhaust particle chemicals in bronchial epithelial cells and macrophages J Immunol 2002 169 4531 4541 12370390 10.4049/jimmunol.169.8.4531

[B148] Boland S Baeza-Squiban A Fournier T Houcine O Gendron MC Chevrier M Jouvenot G Coste A Aubier M Marano F Diesel exhaust particles are taken up by human airway epithelial cells in vitro and alter cytokine production Am J Physiol 1999 276 L604 L613 10198358 10.1152/ajplung.1999.276.4.L604

[B149] Lim HB Ichinose T Miyabara Y Takano H Kumagai Y Shimojyo N Devalia JL Sagai M Involvement of superoxide and nitric oxide on airway inflammation and hyperresponsiveness induced by diesel exhaust particles in mice Free Radic Biol Med 1998 25 635 644 10.1016/S0891-5849(98)00073-2 9801062

[B150] Li YJ Kawada T Matsumoto A Azuma A Kudoh S Takizawa H Sugawara I Airway inflammatory responses to oxidative stress induced by low-dose diesel exhaust particle exposure differ between mouse strains Exp Lung Res 2007 33 227 244 10.1080/01902140701481062 17620185

[B151] Ichinose T Furuyama A Sagai M Biological effects of diesel exhaust particles (DEP). II. Acute toxicity of DEP introduced into lung by intratracheal instillation Toxicology 1995 99 153 167 10.1016/0300-483X(94)03013-R 7541919

[B152] Chung KF Cytokines in chronic obstructive pulmonary disease Eur Respir J Suppl 2001 34 50s 59s 10.1183/09031936.01.00229701 12392035

[B153] D'hulst AI Maes T Bracke KR Demedts IK Tournoy KG Joos GF Brusselle GG Cigarette smoke-induced pulmonary emphysema in scid-mice. Is the acquired immune system required? Respir Res 2005 6 147 10.1186/1465-9921-6-147 16359546 PMC1334210

[B154] Bracke KR D'hulst AI Maes T Moerloose KB Demedts IK Lebecque S Joos GF Brusselle GG Cigarette smoke-induced pulmonary inflammation and emphysema are attenuated in CCR6-deficient mice J Immunol 2006 177 4350 4359 16982869 10.4049/jimmunol.177.7.4350

[B155] D'hulst AI Bracke KR Maes T De Bleecker JL Pauwels RA Joos GF Brusselle GG Role of tumour necrosis factor-alpha receptor p75 in cigarette smoke-induced pulmonary inflammation and emphysema Eur Respir J 2006 28 102 112 10.1183/09031936.06.00059305 16540505

[B156] Salvi SS Nordenhall C Blomberg A Rudell B Pourazar J Kelly FJ Wilson S Sandstrom T Holgate ST Frew AJ Acute exposure to diesel exhaust increases IL-8 and GRO-alpha production in healthy human airways Am J Respir Crit Care Med 2000 161 550 557 10673199 10.1164/ajrccm.161.2.9905052

[B157] Nordenhall C Pourazar J Blomberg A Levin JO Sandstrom T Adelroth E Airway inflammation following exposure to diesel exhaust: a study of time kinetics using induced sputum Eur Respir J 2000 15 1046 1051 10.1034/j.1399-3003.2000.01512.x 10885423

[B158] Gowdy K Krantz QT Daniels M Linak WP Jaspers I Gilmour MI Modulation of pulmonary inflammatory responses and antimicrobial defenses in mice exposed to diesel exhaust Toxicol Appl Pharmacol 2008 229 310 319 10.1016/j.taap.2008.01.040 18343473

[B159] Hiramatsu K Azuma A Kudoh S Desaki M Takizawa H Sugawara I Inhalation of diesel exhaust for three months affects major cytokine expression and induces bronchus-associated lymphoid tissue formation in murine lungs Exp Lung Res 2003 29 607 622 10.1080/01902140390240140 14594659

[B160] Saber AT Jacobsen NR Bornholdt J Kjaer SL Dybdahl M Risom L Loft S Vogel U Wallin H Cytokine expression in mice exposed to diesel exhaust particles by inhalation. Role of tumor necrosis factor Part Fibre Toxicol 2006 3 4 10.1186/1743-8977-3-4 16504008 PMC1402318

[B161] Fujimaki H Kurokawa Y Yamamoto S Satoh M Distinct requirements for interleukin-6 in airway inflammation induced by diesel exhaust in mice Immunopharmacol Immunotoxicol 2006 28 703 714 10.1080/08923970601067433 17190745

[B162] Churg A Zhou S Wang X Wang R Wright JL The role of interleukin-1beta in murine cigarette smoke-induced emphysema and small airway remodeling Am J Respir Cell Mol Biol 2009 40 482 490 10.1165/rcmb.2008-0038OC 18931327

[B163] Doz E Noulin N Boichot E Guenon I Fick L Le BM Lagente V Ryffel B Schnyder B Quesniaux VF Couillin I Cigarette smoke-induced pulmonary inflammation is TLR4/MyD88 and IL-1R1/MyD88 signaling dependent J Immunol 2008 180 1169 1178 18178857 10.4049/jimmunol.180.2.1169

[B164] Diaz-Sanchez D Tsien A Casillas A Dotson AR Saxon A Enhanced nasal cytokine production in human beings after in vivo challenge with diesel exhaust particles J Allergy Clin Immunol 1996 98 114 123 10.1016/S0091-6749(96)70233-6 8765825

[B165] Kongerud J Madden MC Hazucha M Peden D Nasal responses in asthmatic and nonasthmatic subjects following exposure to diesel exhaust particles Inhal Toxicol 2006 18 589 594 10.1080/08958370600743027 16864550

[B166] Behndig AF Mudway IS Brown JL Stenfors N Helleday R Duggan ST Wilson SJ Boman C Cassee FR Frew AJ Kelly FJ Sandstrom T Blomberg A Airway antioxidant and inflammatory responses to diesel exhaust exposure in healthy humans Eur Respir J 2006 27 359 365 10.1183/09031936.06.00136904 16452593

[B167] Ohtoshi T Takizawa H Okazaki H Kawasaki S Takeuchi N Ohta K Ito K Diesel exhaust particles stimulate human airway epithelial cells to produce cytokines relevant to airway inflammation in vitro J Allergy Clin Immunol 1998 101 778 785 10.1016/S0091-6749(98)70307-0 9648705

[B168] Devalia JL Bayram H Abdelaziz MM Sapsford RJ Davies RJ Differences between cytokine release from bronchial epithelial cells of asthmatic patients and non-asthmatic subjects: effect of exposure to diesel exhaust particles Int Arch Allergy Immunol 1999 118 437 439 10.1159/000024157 10224468

[B169] Bayram H Devalia JL Khair OA Abdelaziz MM Sapsford RJ Sagai M Davies RJ Comparison of ciliary activity and inflammatory mediator release from bronchial epithelial cells of nonatopic nonasthmatic subjects and atopic asthmatic patients and the effect of diesel exhaust particles in vitro J Allergy Clin Immunol 1998 102 771 782 10.1016/S0091-6749(98)70017-X 9819294

[B170] Hirota R Akimaru K Nakamura H In vitro toxicity evaluation of diesel exhaust particles on human eosinophilic cell Toxicol In Vitro 2008 22 988 994 10.1016/j.tiv.2008.02.004 18359185

[B171] Vlahos R Bozinovski S Hamilton JA Anderson GP Therapeutic potential of treating chronic obstructive pulmonary disease (COPD) by neutralising granulocyte macrophage-colony stimulating factor (GM-CSF) Pharmacol Ther 2006 112 106 115 10.1016/j.pharmthera.2006.03.007 16716406

[B172] Ohta K Yamashita N Tajima M Miyasaka T Nakano J Nakajima M Ishii A Horiuchi T Mano K Miyamoto T Diesel exhaust particulate induces airway hyperresponsiveness in a murine model: essential role of GM-CSF J Allergy Clin Immunol 1999 104 1024 1030 10.1016/S0091-6749(99)70084-9 10550748

[B173] Krisiukeniene A Babusyte A Stravinskaite K Lotvall J Sakalauskas R Sitkauskiene B Smoking affects eotaxin levels in asthma patients J Asthma 2009 46 470 476 10.1080/02770900902846349 19544167

[B174] Takizawa H Abe S Okazaki H Kohyama T Sugawara I Saito Y Ohtoshi T Kawasaki S Desaki M Nakahara K Yamamoto K Matsushima K Tanaka M Sagai M Kudoh S Diesel exhaust particles upregulate eotaxin gene expression in human bronchial epithelial cells via nuclear factor-kappa B-dependent pathway Am J Physiol Lung Cell Mol Physiol 2003 284 L1055 L1062 12576300 10.1152/ajplung.00358.2002

[B175] Diaz-Sanchez D Jyrala M Ng D Nel A Saxon A In vivo nasal challenge with diesel exhaust particles enhances expression of the CC chemokines rantes, MIP-1alpha, and MCP-3 in humans Clin Immunol 2000 97 140 145 10.1006/clim.2000.4921 11027454

[B176] Ying S O'Connor B Ratoff J Meng Q Fang C Cousins D Zhang G Gu S Gao Z Shamji B Edwards MJ Lee TH Corrigan CJ Expression and cellular provenance of thymic stromal lymphopoietin and chemokines in patients with severe asthma and chronic obstructive pulmonary disease J Immunol 2008 181 2790 2798 18684970 10.4049/jimmunol.181.4.2790

[B177] Bratke K Klug M Bier A Julius P Kuepper M Virchow JC Lommatzsch M Function-associated surface molecules on airway dendritic cells in cigarette smokers Am J Respir Cell Mol Biol 2008 38 655 660 10.1165/rcmb.2007-0400OC 18203971

[B178] Lommatzsch M Bratke K Knappe T Bier A Dreschler K Kuepper M Stoll P Julius P Virchow JC Acute effects of tobacco smoke on human airway dendritic cells in vivo Eur Respir J 2009 Sep 9, [Epub ahead of print] 19741025 10.1183/09031936.00090109

[B179] Demedts IK Bracke KR Van Pottelberge G Testelmans D Verleden GM Vermassen FE Joos GF Brusselle GG Accumulation of dendritic cells and increased CCL20 levels in the airways of patients with chronic obstructive pulmonary disease Am J Respir Crit Care Med 2007 175 998 1005 10.1164/rccm.200608-1113OC 17332482

[B180] D'hulst AI Vermaelen KY Brusselle GG Joos GF Pauwels RA Time course of cigarette smoke-induced pulmonary inflammation in mice Eur Respir J 2005 26 204 213 10.1183/09031936.05.00095204 16055867

[B181] Robbins CS Franco F Mouded M Cernadas M Shapiro SD Cigarette smoke exposure impairs dendritic cell maturation and T cell proliferation in thoracic lymph nodes of mice J Immunol 2008 180 6623 6628 18453581 10.4049/jimmunol.180.10.6623 PMC2885874

[B182] Freeman CM Martinez FJ Han MK Ames TM Chensue SW Todt JC Arenberg DA Meldrum CA Getty C McCloskey L Curtis JL Lung Dendritic Cell Expression of Maturation Molecules Increases with Worsening COPD Am J Respir Crit Care Med 2009 180 12 1179 1188 10.1164/rccm.200904-0552OC 19729666 PMC2796731

[B183] Thomson NC Chaudhuri R Livingston E Asthma and cigarette smoking Eur Respir J 2004 24 822 833 10.1183/09031936.04.00039004 15516679

[B184] Gorska K Maskey-Warzechowska M Krenke R Airway inflammation in chronic obstructive pulmonary disease Curr Opin Pulm Med 2009 Nov 9, [Epub ahead of print] 19904205 10.1097/MCP.0b013e3283341ba0

[B185] Terada N Maesako K Hiruma K Hamano N Houki G Konno A Ikeda T Sai M Diesel exhaust particulates enhance eosinophil adhesion to nasal epithelial cells and cause degranulation Int Arch Allergy Immunol 1997 114 167 174 10.1159/000237663 9338611

[B186] Nightingale JA Maggs R Cullinan P Donnelly LE Rogers DF Kinnersley R Chung KF Barnes PJ Ashmore M Newman-Taylor A Airway inflammation after controlled exposure to diesel exhaust particulates Am J Respir Crit Care Med 2000 162 161 166 10903236 10.1164/ajrccm.162.1.9908092

[B187] Stanescu D Sanna A Veriter C Kostianev S Calcagni PG Fabbri LM Maestrelli P Airways obstruction, chronic expectoration, and rapid decline of FEV1 in smokers are associated with increased levels of sputum neutrophils Thorax 1996 51 267 271 10.1136/thx.51.3.267 8779129 PMC1090637

[B188] Salvi S Blomberg A Rudell B Kelly F Sandstrom T Holgate ST Frew A Acute inflammatory responses in the airways and peripheral blood after short-term exposure to diesel exhaust in healthy human volunteers Am J Respir Crit Care Med 1999 159 702 709 10051240 10.1164/ajrccm.159.3.9709083

[B189] Diaz-Sanchez D Dotson AR Takenaka H Saxon A Diesel exhaust particles induce local IgE production in vivo and alter the pattern of IgE messenger RNA isoforms J Clin Invest 1994 94 1417 1425 10.1172/JCI117478 7523450 PMC295270

[B190] Warr GA Martin RR Sharp PM Rossen RD Normal human bronchial immunoglobulins and proteins: effects of cigarette smoking Am Rev Respir Dis 1977 116 25 30 879597 10.1164/arrd.1977.116.1.25

[B191] Tsoumakidou M Elston W Zhu J Wang Z Gamble E Siafakas NM Barnes NC Jeffery PK Cigarette smoking alters bronchial mucosal immunity in asthma Am J Respir Crit Care Med 2007 175 919 925 10.1164/rccm.200607-908OC 17303795

[B192] Di Benedetto G Passive smoking in childhood J R Soc Health 1995 115 13 16 10.1177/146642409511500105 7738974

[B193] Mamessier E Nieves A Vervloet D Magnan A Diesel exhaust particles enhance T-cell activation in severe asthmatics Allergy 2006 61 581 588 10.1111/j.1398-9995.2006.01056.x 16629788

[B194] Broekema M ten Hacken NH Volbeda F Lodewijk ME Hylkema MN Postma DS Timens W Airway Epithelial Changes in Smoking but not in Ex-Smoking Asthmatics Am J Respir Crit Care Med 2009 180 12 1170 1178 10.1164/rccm.200906-0828OC 19797761

[B195] Takizawa R Pawankar R Yamagishi S Takenaka H Yagi T Increased expression of HLA-DR and CD86 in nasal epithelial cells in allergic rhinitics: antigen presentation to T cells and up-regulation by diesel exhaust particles Clin Exp Allergy 2007 37 420 433 10.1111/j.1365-2222.2007.02672.x 17359392 PMC7164828

[B196] Diaz-Sanchez D Penichet-Garcia M Saxon A Diesel exhaust particles directly induce activated mast cells to degranulate and increase histamine levels and symptom severity J Allergy Clin Immunol 2000 106 1140 1146 10.1067/mai.2000.111144 11112898

