# Supplementary material for: Mouse models to unravel the role of inhaled pollutants on allergic sensitization and airway inflammation
Source: Respir Res. 2010 Jan 21;11(1):7. doi: 10.1186/1465-9921-11-7 (PMC2831838; doi:10.1186/1465-9921-11-7)
Supplement: Additional file 1 — Table 2: Effects of environmental tobacco smoke (ETS) on murine allergic sensitization. Table 2 provides a detailed overview of methodologies and results from murine models that examine the effects of ETS on allergic sensitization [file 1465-9921-11-7-S1.PDF]

Additional file 1: Table 2: EFFECTS OF **ENVIRONMENTAL TOBACCO SMOKE (ETS)** ON **MURINE ALLERGIC SENSITIZATION**

**Mice**	**Sensitization**	**Exposure protocol**	**Immunoglobulins**	**Inflammation**	**Airway responsiveness or remodeling features**	**Reference**
Female BALB/c or C57BL/6	No	OVA aerosol, ETS or OVA/ETS for 10 days<br><br>Rechallenge : OVA aerosol at day 30	C57BL/6: OVA- IgE ↑ and OVA-IgG_1 ↑ in OVA/ETS group at days 12, 18, 25 and 30, but not in OVA group<br><br>BALB/c: similar to C57BL/6, but transient increase in OVA-IgE in OVA group	BAL total cell numbers and eosinophils ↑ in OVA/ETS compared to OVA or ETS alone in both strains.<br><br>In C57BL/6: neutrophil numbers ↑ in OVA/ETS group<br><br>BAL IL-5 ↑ , GM-CSF ↑ and IL-2 ↑ and IFNγ ↓ in OVA/ETS compared to OVA	N.D.	Rumold et al, 2001 [63]
Female BALB/c	i.p. OVA-sensitized (alum)	ETS or air from day 1 to 43 Single OVA or PBS aerosol on day 17	Total IgE ↑ and OVA-IgG_1 ↑ in OVA/ETS compared to OVA/air.	Blood eosinophilia ↑ in OVA/ETS group compared to OVA/air<br><br>Lung eosinophils and lymphocytes ↑ in OVA/ETS group compared to OVA/air<br><br>OVA-stimulated lung cells: IL-4 ↑ and IL-10 ↑ in OVA/ETS compared to OVA/air	N.D.	Seymour at al, 1997 [64]
Males/females BALB/c	i.p. OVA-sensitized (alum)	ETS or air in utero until day 80 after birth. OVA aerosol on day 14, 28 and 80.	Total IgE ↑,OVA-IgE ↑ and IgG_1 ↑ in females compared to in males.<br><br>OVA-IgE ↑ in OVA/ETS in females.<br>OVA-IgG_1 ↑ in OVA/ETS in males	Number of IgE positive cells in lung parenchyma ↑ in OVA/ETS in females, not in males<br><br>Blood eosinophilia ↑ by ETS (independent of sex). After OVA aerosol, eosinophils ↑ more in females<br><br>OVA-stimulated lung cells: higher levels of Th2 cytokines (IL-13, IL-5, and IL-10) in females. Enhancing effects of ETS more apparent for males.	N.D.	Seymour et al, 2002 [65]
Male DO11.10 OVA-T cell receptor hemizygous mice (+/-)	No	Postnatal 4 weeks air, followed by 6 weeks ETS or air, combined with OVA	OVA-IgE ↑ and IgG_1 ↑ upon OVA, trend to be reduced upon OVA/ETS	BAL lymphocytes ↑ and neutrophils ↑ in OVA and OVA/ETS group, slight increase in eosinophils in both groups	AHR ↑ in OVA/ETS group compared to ETS or air.<br><br>Occasional mucous cell hyperplasia in OVA and OVA/ETS group	Barret et al, 2002 [66]
Female C57BL/6, BALB/c and A/J	No	Whole body exposure to ETS or filtered air for 9 weeks, combined with nose-only aerosol (10 days) of saline or OVA on weeks 2 and 3; 5 and 6 or 8 and 9	No effect of ETS	A/J: moderate inflammation which ↓ by ETS<br>BALB/c : limited inflammation, no effect of ETS<br>C57BL/6: no inflammation towards OVA, no effect of ETS.	No effects of OVA or ETS on AHR	Bowles et al, 2005 [67]
Males/Females BALB/c	No	In utero ETS or Air, OVA or saline aerosol in weeks 7 and 8 after birth	No effect of ETS	No effect of ETS	AHR ↑ in OVA/ETS compared to OVA/air	Penn et al, 2007 [68]
Female BALB/c	i.n. sensitization to Af	ETS or air for 43 days, combined with 4 i.n. Af-applications (days 14, 17, 21, 24)	No effect of ETS	Blood eosinophilia ↑ in Af/ETS compared to Af /air<br><br>No difference in cytokine responses from homogenized lung.	AHR ↑ in Af/ETS compared to Af/air	Seymour et al, 2003 [69]

OVA: ovalbumin, ETS: environmental tobacco smoke, BAL: bronchoalveolar lavage fluid, Af: Aspergillus fumigatus, OVA-Ig: OVA-specific immunoglobulin, Af-Ig: Af-specific immunoglobulin
i.p. intraperitoneal, i.n.: intranasal, AHR: airway hyperresponsiveness, N.D.: not determined
