# Supplementary material for: Mouse models to unravel the role of inhaled pollutants on allergic sensitization and airway inflammation
Source: Respir Res. 2010 Jan 21;11(1):7. doi: 10.1186/1465-9921-11-7 (PMC2831838; doi:10.1186/1465-9921-11-7)
Supplement: Additional file 2 — Table 3: Effects of mainstream cigarette smoke (MS) on murine allergic sensitization. Table 3 provides a detailed overview of methodologies and results from murine models that examine the effects of MS on allergic sensitization [file 1465-9921-11-7-S2.PDF]

Additional file 2: Table 3: EFFECTS OF **MAINSTREAM CIGARETTE SMOKE (MS)** ON **MURINE ALLERGIC SENSITIZATION**

Mice	Sensitization and mice	Exposure protocol	Immunoglobulins	Inflammation	Airway responsiveness or remodeling features	Reference
Female BALB/c	OVA-specific mucosal sensitisation (with GMCSF- adenoviral construct and OVA-aerosol for 10 days)	MS or air exposure for 2-3 months, followed by the sensitization protocol<br><br>For AHR measurement: OVA rechallenge 4 weeks after last OVA exposure	No effects on OVA-IgE and OVA-IgG_1, but OVA-IgG_{2a} ↓	OVA-stimulated spleen cells: IL-4 ↑ and IL-5 ↑ in OVA/MS compared to OVA/air<br><br>BAL eosinophils ↓ and neutrophils ↓ in OVA/MS compared to OVA/air<br>Lung dendritic cells ↓, T-cells ↓ and B-cells ↓ in OVA/MS compared to OVA/air<br><br>Trend towards IL-5 ↑, IL-13 ↑ and eoxtaxin ↑ in BAL and IL-13 ↑ in serum in OVA/MS compared to OVA/air	AHR ↓ in OVA/MS compared to OVA/air	Robbins et al 2005 [70]
Male BALB/c	i.n. RW for 7 days	MS for 5 weeks, with in the final week i.n. RW applications	No effect on RW-IgG_1 production	BAL and lung eosinophils ↓ in MS/RW compared to the RW-only<br><br>RW-stimulated spleen cells: IL-4 ↑, IL-5 ↑ and IL-13 ↑ in MS/RW compared RW-only	Goblet cells ↓ in MS/RW to RW-only	Robbins et al 2005 [70]
Male BALB/c	No	Daily OVA or PBS aerosol, combined with air or MS for 3 weeks, (exposures simultaneous).	OVA-IgE ↑ and OVA-IgG_1 ↑ in OVA/MS compared to OVA/air	BAL/lung eosinophils ↑ , dendritic cells ↑ and lymphocytes ↑ in OVA/MS, not in OVA/air<br><br>TARC ↑ and IFNg ↑ in BAL in OVA/MS<br><br>OVA-stimulated lymph node cells: IL-5 ↑ in OVA/ MS compared to OVA/air	Goblet cells ↑ in OVA/MS, not in OVA/air<br><br>No effect of OVA/MS on AHR	Moerloose et al 2006 [71]<br><br>Robays et al 2009 [72]
Female BALB/c	No	Daily OVA aerosol, combined with air or MS for 2 weeks<br><br>Rechallenge experiment after 4 weeks	N.D.<br><br>OVA-IgG_1 ↑, OVA-IgE ↑ and OVA-IgG_{2a}↑ in OVA/MS compared to all groups	Pulmonary eosinophils ↑ and $CD69^+$-T-cells ↑ in OVA/MS, not in OVA/air. Neutrophilia ↓ in OVA/MS compared to MS alone<br><br>Pulmonary mononuclear cells ↑, eosinophils ↑, dendritic cells ↑, $CD69^+$-T-cells ↑ and IL-5+ T-cells ↑ in OVA/MS group compared to all other groups	Goblet cell ↑ in OVA/MS, not in other groups<br><br>Goblet cell ↑ in OVA/MS, not in other groups	Trimble et al 2009 [73]

OVA: ovalbumin, RW: ragweed, MS: mainstream cigarette smoke, BAL: Bronchoalveolar lavage fluid, i.n.: intranasal, AHR: airway hyperresponsiveness, OVA-Ig: OVA-specific immunoglobulin, RW-Ig: RW-specific immunoglobulin, N.D. not determined
