# Supplementary material for: Mouse models to unravel the role of inhaled pollutants on allergic sensitization and airway inflammation
Source: Respir Res. 2010 Jan 21;11(1):7. doi: 10.1186/1465-9921-11-7 (PMC2831838; doi:10.1186/1465-9921-11-7)
Supplement: Additional file 3 — Table 4: Effects of diesel exhaust particles (DEP) on murine allergic sensitization. Table 4 provides a detailed overview of methodologies and results from murine models that examine the effects of DEP on allergic sensitization [file 1465-9921-11-7-S3.PDF]

Additional file 3: Table4: EFFECTS OF **DIESEL EXHAUST PARTICLES (DEP)** ON **MURINE ALLERGIC SENSITIZATION**

**Mice**	**Sensitisation / Exposure**	**Immunoglobulins**	**Inflammation**	**Airway responsiveness or remodeling features**	**Reference**
Female BDF1	1 x i.p. OVA or OVA+DEP (no alum)<br><br>5 x i.p. DNP-OVA or DNP-OVA+DEP at 3-week intervals<br><br>5 x i.p JCP or JCP+DEP at 4-week intervals	OVA-IgE ↑in OVA+DEP compared to OVA alone<br><br>DNP-OVA-IgE ↑ in DNP-OVA+DEP compared to DNP-OVA alone<br><br>JCP-IgE ↑ in JCP+DEP compared to JCP alone	N.D.	N.D.	Muranaka et al 1986 [74]
Female BDF1	5 x i.n. OVA or OVA+DEP at 3-week intervals<br><br>9 x i.n. OVA or OVA+DEP at 1-week interval	OVA-IgE ↑ in OVA+DEP compared to OVA alone	N.D.	N.D.	Takafuji et al, 1987 [75]
Male BALB/c	3 x i.t. OVA or OVA+DEP, JCP or JCP+DEP at 3-week intervals	OVA-IgE ↑ and JCP-IgE ↑ in OVA+DEP and JCP+DEP respectively compared to OVA alone	After OVA or JCP restimulation of mediastinal LN cells: proliferation ↑, IL-2 ↑ and IL-4 ↑ in OVA+DEP and JCP+DEP compared to OVA or JCP alone	N.D.	Fujimaki et al, 1994 [76]
Male BALB/c	3 x i.n. OVA or OVA+DEP at 3-week intervals	Trend to OVA-IgE ↑ in OVA+DE compared to OVA+air	After OVA restimulation of cervical LNcells: Proliferation ↑, IFN-γ ↓ and IL-4 ↑ in OVA+DEP compared to OVA alone	N.D.	Fujimaki et al, 1995 [77]
Male BALB/c	3 x i.n. OVA at 3-week interval<br><br>3 weeks inhalation of DE or air	OVA-IgE ↑ in OVA+DE compared to OVA+air (no effect on total IgE and OVA-IgG)	Spleen weight ↑ in OVA+DE compared to OVA+air<br><br>After OVA restimulation of spleen cells: IFN-γ ↓, IL-4↑ and IL-10↑ in OVA+DE compared to OVA+air	N.D.	Fujimaki 1997 [78]
Female BALB/c	1 x s.c. in footpad OVA or OVA+DEP	OVA-IgE ↑ in OVA+DEP compared to OVA alone	Popliteal LN weight ↑, cell numbers ↑ and cell proliferation ↑ in OVA+DEP compared to OVA alone	N.D.	Lovik et al 1997 [79]
Female BALB/cA	4 x i.n. OVA or OVA+DEP at week 0, 2, 4 and 5	OVA-IgE ↑ in OVA+DEP compared to OVA alone		N.D.	Nilsen et al 1997 [80]
Female BDF1	3 x i.p. OVA or OVA+DEP at 1-week interval (OVA/alum as positive control)	OVA-IgE ↑,OVA- IgG_1 ↑ and OVA-IgG_{2a} ↑ in OVA+DEP compared to OVA alone<br><br>Total IgE ↑ and IgG_1 ↑ in OVA+DEP compared to OVA alone (no effect on total IgG_{2a})	N.D.	N.D.	Heo et al 2001 [81]
Not specified	7 x i.n. Der f II or Der f II+DEP at 2-week intervals	Der f II-IgE ↑ and IgG_1 ↑ in Der f II+DEP compared to Der f II alone	N.D.	N.D.	Suzuki et al 1996 [82]
BDF1, ICR, C57BL/6 C3H/He, CBA/2N	4 x i.t. OVA or OVA+DEP at 3-week intervals (no alum)	OVA-IgG_1 ↑ in BDF1 in OVA+DEP compared to OVA alone (no effect on OVA-IgE)	Lung eosinophils ↑ in CBA/2N, BDF1, ICR and C57BL/6 in OVA+DEP group compared to OVA alone	Goblet cells ↑ in BDF1, ICR and C57BL/6 in OVA+DEP compared to OVA alone	Ichinose et al, 1997 [83]
Female BALB/c	1 x s.c. in footpad TNP-OVA + DEP or saline (primary response), after 4 weeks 1 x i.n. TNP-OVA (secondary response)	Primary response: TNP-IgG_1 ↑ in DEP vs saline<br>Secondary reponse: TNP-IgG_1 ↑ and TNP-IgE ↑ in DEP vs saline	Primary response: popliteal LN B cells ↑, CD86 and CD40 expression on B-cells ↑, IL-4^+CD4^+ cells ↑, $IFN_\gamma^+CD4^+$ cells ↑ , $IFN_\gamma^+CD8^+$ cells in DEP vs saline<br><br>Secondary response: number of TNP-IgG_1 and TNP-IgE forming cells ↑ in peribronchial LN and spleen	N.D.	Van Zijverden et al 2000 [84]

C57BL/6N and CBA/JN	4 x i.t. Der f or Der f+DEP at 2-week intervals	Der f-IgG_1↑ in C57BL/6N and CBA/JN in Der f+DEP compared to Der f alone (no effect on Der f-IgE)	Lung eosinophils ↑ and lymphocytes ↑ in CBA/JN in Der f+DEP compared to Der f alone<br><br>Lung levels of eotaxin ↑ and GM-CSF ↑ in C57BL/6N and IL-5 ↑, eotaxin ↑and RANTES ↑ in CBA/JN in Der f+DEP compared to Der f alone (no effect on IL-2 and IL-4)	Goblet cells ↑ in C57BL/6N and CBA/JN in Der f+DEP compared to Der f alone	Sadakane et al, 2002 [85]
Female BALB/c	10 x daily inhalation OVA or OVA+DEP (no alum)	OVA-IgE ↑, OVA-IgG_1↑ and Total IgE ↑ in OVA+DEP compared to OVA alone	Only neurophilia by DEP	No mucin production in this model	Whitekus et al, 2002 [86]
Male BALB/c	2 x i.n. OVA or OVA+DEP at days 0 and 14, followed by three OVA challenges (i.n. or aerosol) with or without DEP at days 35, 38 and 41	OVA-IgE ↑ in OVA+DEP compared to OVA alone with both challenge protocols	Peribronchial and perivascular infiltrates ↑ and eosinophils ↑ in OVA+DEP compared to OVA alone	Goblet cells ↑ in OVA+DEP compared to OVA alone	Steerenberg et al, 2003 [87]
BALB/c, ICR, C3H/HeN	4 x i.t. Der f or Der f+DEP at 2-week intervals	Der f-IgG_1↑ in C3H/He in Der f+DEP compared to Der f alone	Lung eosinopils ↑, lung IL-5 ↑ and eotaxin ↑ in Der f+DEP compared to Der f alone	Goblet cells ↑ in Der f +DEP compared to Der f	Ichinose et al, 2004 [88]

OVA: Ovalbumin, DEP: diesel exhaust particles, DNP-OVA:Dinitrophenylated-OVA, JCP: Japanese Cedar Pollen, Der f: Dermatophagoides farinae, BAL: Bronchoalveolar lavage fluid, OVA-Ig: OVA-specific immunoglobulin, DNP-OVA-Ig: DNP-OVA-specific immunoglobulin, JCP-Ig:JCP-specific immunoglobulin, TNP-OVA: 2, 4, 6-trinitrophenyl coupled to ovalbumin, TNP-Ig: TNP-specific immunoglobulin, Der f-Ig: Der f-specific immunoglobulin, i.p.: intraperitoneal, i.n.: intranasal, i.t: intratracheal, LN: lymph node, N.D.: not determined
