# Supplementary material for: Mouse models to unravel the role of inhaled pollutants on allergic sensitization and airway inflammation
Source: Respir Res. 2010 Jan 21;11(1):7. doi: 10.1186/1465-9921-11-7 (PMC2831838; doi:10.1186/1465-9921-11-7)
Supplement: Additional file 4 — Table 5: Effects of environmental tobacco smoke (ETS) on development or aggravation of asthma in murine models. Table 5 provides a detailed overview of methodologies and results from murine models that examine the effects of ETS on development or aggravation of asthma [file 1465-9921-11-7-S4.PDF]

Additional file 4: Table 5: EFFECTS OF **ENVIRONMENTAL TOBACCO SMOKE** ON **DEVELOPMENT OR AGGRAVATION** OF ASTHMA IN MURINE MODELS

**Mice**	**Sensitization**	**Exposure protocol**	**Immunoglobulins**	**Inflammation**	**Airway responsiveness or remodeling features**	**Reference**
Female BALB/c	i.p. OVA-sensitized (+alum)	ETS or air from day 1 to 43 combined with single OVA or PBS aerosol on day 17	Total IgE ↑ and IgG_1 ↑ in OVA/ETS compared to OVA/air.	Blood eosinophilia ↑ in OVA/ETS compared to OVA/air<br><br>Lung eosinophils and lymphocytes ↑ in OVA/ETS compared to OVA/air<br><br>OVA-stimulated lung cells: IL-4 ↑ and IL-10 ↑ in OVA/ETS compared to OVA/air	N.D.	Seymour at al, 1997 [64]
Males/ Females BALB/c	i.p. OVA-sensitized (+alum)	ETS or air in utero until day 80 after birth. OVA aerosol on day 14, 28 and 80.	Total IgE ↑, OVA-IgE ↑ and IgG_1 ↑ in females compared to males.<br><br>OVA-IgE ↑ in OVA/ETS in females.<br>OVA-IgG_1 ↑ in OVA/ETS in males	Number of IgE positive cells in lung parenchyma ↑ in OVA/ETS in females, not in males<br><br>Blood eosinophilia ↑ by ETS (independent of sex). After OVA aerosol, eosinophils ↑ more in females<br><br>OVA-stimulated lung cells: higher levels of Th2 cytokines (IL-13, IL-5, and IL-10) in females. Enhancing effects of ETS more apparent for males.	N.D.	Seymour et al 2002 [65]
Males/ Females BALB/c	s.c. OVA on days 0, 7, 14, and 21 (+alum)	i.n. OVA challenges on days 27, 29, 31 and twice weekly during 4 weeks, with or without ETS		BAL and peribronchial eosinophils ↑ in OVA/ETS compared to OVA alone<br><br>BAL IL-5 ↑ in OVA/ETS compared to OVA alone<br><br>BAL and airway epithelial eotaxin-1 ↑ in OVA/ETS compared to OVA alone	Smooth muscle thickness ↑, α-smooth muscle actine ↑, peribronchial collagen ↑, TGF-β positive peribronchial cells ↑ in OVA/ETS compared to OVA alone<br><br>AHR ↑ in OVA/ETS compared to OVA alone<br><br>PAS-positive and Alcian blue-positive cells ↑ in OVA/ETS compared to OVA alone	Min et al, 2007 [89]
Males/ Females BALB/c	i.p. sensitization (+alum)	In utero exposure to ETS or air,<br>OVA or saline aerosol in weeks 7-8,<br>i.p. sensitization in weeks 11 and 13, OVA aerosol in weeks 14 and 15	No effects on immunoglobulin production	Saline aerosol prior to OVA sensitization/challenge: BAL eosinophils ↑ and IL-5 ↑ in mice that were in utero exposed to ETS compared to air<br><br>OVA aerosol prior to OVA sensitization/challenge: no inflammation	Saline aerosol prior to OVAsensitization/challenge: AHR ↑ in mice that were in utero exposed to ETS compared to air<br><br>OVA aerosol prior to OVA sensitization/challenge: AHR ↓ compared to mice that inhaled saline, and AHR ↓↓ in animals that were in utero exposed to ETS	Pen et al 2007 [68]
Males/ Females BALB/c	No	In utero exposure to MS or air<br>i.n. Der p or PBS from week 5-10 after birth	No effect of maternal smoking on IgE	Neutrophils ↑ and mast cells ↑ in Der p/MS compared to Der p/air<br><br>No effect of maternal smoking on lung cytokine production	Goblet cells ↑ in Der p/MS compared to Der p/air<br><br>Maternal smoking: airway smooth muscle layer ↑, collagen III ↑ and AHR ↑, independent of Der p	Blacquière et al, 2009 [90]

OVA: ovalbumin, ETS: environmental tobacco smoke, MS: mainstream cigarette smoke, Der p: Dermatophagoides pteronyssinus, BAL: bronchoalveolar lavage fluid, OVA-Ig: OVA-specific immunoglobulin, i.p. : intraperitoneal, i.n.: intranasal, s.c.: subcutaneous, i.t: intratracheal, AHR: airway hyperresponsiveness, N.D.: not determined
