# Supplementary material for: Mouse models to unravel the role of inhaled pollutants on allergic sensitization and airway inflammation
Source: Respir Res. 2010 Jan 21;11(1):7. doi: 10.1186/1465-9921-11-7 (PMC2831838; doi:10.1186/1465-9921-11-7)
Supplement: Additional file 5 — Table 6: Effects of mainstream cigarette smoke (MS) on development or aggravation of asthma in murine models. Table 6 provides a detailed overview of methodologies and results from murine models that examine the effects of MS on development or aggravation of asthma [file 1465-9921-11-7-S5.PDF]

Additional file 5: Table 6: EFFECTS OF **MAINSTREAM CIGARETTE SMOKE** ON **DEVELOPMENT OR AGGRAVATION** OF ASTHMA IN MURINE MODELS

| **Mice** | **Sensitisation** | **Exposure protocol** | **Immunoglobulins** | **Inflammation** | **Airway responsiveness or remodeling features** | **Reference** |
|---|---|---|---|---|---|---|
| Male/female BALB/c | No | In utero exposure to air or whole body MS, followed by ETS or air exposure for 10 weeks<br>Single i.t. injection of Af extract (Af), 48 hrs before AHR measurement<br><br>MS exposure of adult mice for 10 weeks, followed by i.t. injection of Af extract | N.D. | Af induces eosinophilia in BAL, but no effect of prenatal exposure<br><br>Lung cyclic AMP levels ↓ and phosphodiesterase-4 activity ↑ in mice prenatal exposed to MS<br><br>No effect on cyclic AMP levels and phosphodiesterase- 4 activity | AHR ↑ in Af treated-mice prenatal exposed to MS, even when postnatal exposed to air for 10 weeks.<br><br>No effect on mucous production<br><br>No effect on AHR towards Af extract | Singh et al , 2003 [91] |
| Male C57Bl/6j | i.p. OVA-sensitized (+alum) | OVA or PBS aerosol for 7 weeks<br>From week 4 to7: combined with MS (nose-only exposure) or air | No effect on total and OVA-IgE | Eosinophils ↓ in BAL in OVA/MS compared to OVA/air<br><br>Lung macrophages ↓ and activated CD8+ T-cells ↓ in OVA/MS compared to OVA/air<br><br>No effect of MS on lung tissue eosinophils, and CD4+ T-cells . | Acute bronchoconstriction ↓ and AHR towards metacholine ↓ in OVA/MS compared to OVA/air | Melgert et al, 2004 [92] |
| Female BALB/c | OVA-specific mucosal sensitisation (with GMCSF-adenoviral construct and OVA-aerosol for 10 days) | MS or air exposure for 2-3 months, followed by the sensitization protocol<br><br>For AHR measurement: OVA rechallenge 4 weeks after last OVA exposure | No effects on OVA-IgE and OVA-$IgG_1$, but OVA-$IgG_{2a}$ ↓ | OVA-stimulated spleen cells: IL-4 ↑ and IL-5 ↑in OVA/MS compared to OVA/air<br><br>BAL eosinophils ↓ and neutrophils ↓ in OVA/MS compared to OVA/air; Lung dendritic cells ↓, T-cells ↓ and B-cells ↓ in OVA/MS compared to OVA/air<br>Trend towards IL-5 ↑, IL-13 ↑ and eoxtaxin ↑ in BAL and IL-13 ↑ in serum in OVA/MS compared to OVA/air | AHR ↓ in OVA/MS compared to OVA/air | Robbins et al 2005 [70] |
| Male BALB/c | i.p. OVA-sensitized (+alum) | OVA or PBS aerosol on days 14, 16, 18, 21 and 24. Air or MS from day 14 until 24 | OVA-IgE ↑ in OVA/MS compared to OVA or smoke alone | Total BAL cells ↑, BAL macrophages ↑, BAL dendritic cells ↑, lung CD4+ ↑, lung CD8+T-cells ↑ and lung dendritic cells ↑ in OVA/MS compared to OVA | AHR towards carbachol in OVA/MS, but not in the OVA/air or PBS/MS groups. | Moerloose et al 2005 [93] |
| Male C57BL/6 | i.p. OVA-sensitization (+alum) | OVA or PBS aerosol from day 14, for 2, 4 or 8 weeks, combined with MS or air | No effect on total and OVA-IgE at all time points | 2 weeks: Th2-inflammation ↑: BAL eosinophils ↑ and lymphocytes ↑, TARC ↑ in BAL, lung dendritic cells ↑ and lung $CD4^+$-T-cells ↑ in OVA/MS group compared to OVA/air<br><br>4 weeks: development of tolerance (reduction of Th2 response) is delayed in OVA/MS group compared to OVA<br><br>8 weeks: No difference between OVA/air and OVA/MS group: inflammation disappears in both groups | N.D. | Van Hove et al 2008, [94] |
| Male C57Bl/6j | i.p. OVA-sensitized (+alum) | OVA or PBS aerosol for 5 months<br>After 1 month of aerosol: combined with air or MS | No increased OVA-IgE in OVA/MS compared to OVA/air, but MS as such increased OVA-IgE levels | OVA-induced Th2-type inflammation is not affected | No effect of MS on OVA-induced goblet cell hyperplasia, airway wall thickness (α-smooth muscle actine) and Collagen III deposition<br><br>No emphysema upon chronic MS, and no effect of the combination OVA/MS | Melgert et al 2007 [95] |

| | | | | | | |
|---|---|---|---|---|---|---|
| Female BALB/c | i.p. OVA-sensitization (+alum) | OVA aerosol on day 5 and 19<br>MS (high or low dose) from day 6 until day of sacrifice. Mice sacrified on days 20, 22 and 26 | High MS dose: delayed production of OVA-IgE, OVA-specific $IgG_1$ ↓ and $IgG_{2a}$ ↓ in OVA/MS group<br><br>Low MS dose: no suppression of OVA-$IgG_1$ and IgE in OVA/MS | High MS dose: eosinophilia ↓, tissue inflammation ↓, IL-4 ↓ and IL-5 ↓ in BAL, loss of antigen-specific proliferation and cytokine production by T-cells in OVA/MS compared to OVA<br><br>Low MS dose: no effect on BAL cells, eosinophilia and neutrophilia | High MS dose: goblet cell metaplasia ↓ in OVA/MS compared to OVA alone | Thatcher et al, 2008, [96] |

OVA: ovalbumin, MS: mainstream cigarette smoke, Af: Aspergillus fumigatus, i.p.: intraperitoneal, i.t: intratracheal, BAL: Bronchoalveolar lavage fluid, AHR: airway hyperresponsiveness, OVA-Ig: OVA-specific immunoglobulin, N.D.: not determined
