# Supplementary material for: Mouse models to unravel the role of inhaled pollutants on allergic sensitization and airway inflammation
Source: Respir Res. 2010 Jan 21;11(1):7. doi: 10.1186/1465-9921-11-7 (PMC2831838; doi:10.1186/1465-9921-11-7)
Supplement: Additional file 6 — Table 7: Effects of diesel exhaust particles (DEP) on development or aggravation of asthma in murine models. Table 7 provides a detailed overview of methodologies and results from murine models that examine the effects of DEP on development or aggravation of asthma [file 1465-9921-11-7-S6.PDF]

Additional file 6: Table 7: EFFECTS OF **DIESEL EXHAUST PARTICLES (DEP)** ON **DEVELOPMENT OR AGGRAVATION** OF ASTHMA IN MURINE MODELS

**Mice**	**Sensitisation**	**Exposure protocol**	**Immunoglobulins**	**Inflammation**	**Airway responsiveness or remodeling features**	**Reference**
Male ICR	3 x i.t. sensitization to OVA or saline at 3-week intervals (no alum)	6 x i.t. DEP or saline at 1-week interval	OVA-IgE ↑, IgG_1 ↑ and IgG_{2a} ↑ in OVA/DEP compared to OVA/saline	BAL and lung eosinophils ↑, BAL neutrophils ↑ and BAL and lung lymphocytes ↑ in OVA+DEP compared to OVA+saline<br><br>Lung: IL-5 ↑ and IL-2 ↑ in OVA+DEP compared to OVA+saline	Goblet cells ↑ in OVA+DEP compared to OVA+saline<br><br>AHR ↑ in OVA+DEP compared to OVA+saline	Takano et al, 1997 [104]<br><br>Takano et al, 1998 [105]
Male ICR and C3H/HeN	i.p. sensitization to OVA(+alum) or saline at day 7	5 weeks inhalation of DE or air<br>OVA inhalation 4 weeks after sensitization	ICR: No effect on OVA-IgE and IgG_1<br><br>C3H/HeN: OVA-IgE ↑ and IgG1 ↑ in OVA+DE compared to OVA+air	ICR and C3H/HeJ: BAL and lung eosinophils ↑ and BAL neutrophils ↑ in OVA+DE compared to OVA+air<br>C3H/HeN: BAL total cells ↑, macrophages ↑ in OVA+DE compared to OVA+air<br><br>ICR: Lung: IL-5 ↑ in OVA+DE group compared to OVA+air<br>C3H/HeN: Lung: IL-5 ↑ and IL-4 ↓ and GM-CSF ↓ in OVA/DE compared to OVA/air	Goblet cells ↑ in OVA+DE compared to OVA+air in both strains<br><br>AHR ↑ in OVA+DE compared to OVA+air in both strains	Miyabara et al, 1998 [106]<br><br>Miyabara et al, 1998 [107]
Male C3H/HeN	i.p. sensitization to OVA or saline at day 0 (no alum)	12 weeks inhalation of DE or air and<br>3 weeks after sensitization, 4 x OVA inhalation at 3-week intervals	OVA-IgE ↑ and IgG_1 ↑ in OVA+DE compared to OVA+air	BAL neutrophils ↑, trend to eosinophils ↑, Lung eosinophils ↑ and mast cells ↑ in OVA+DE compared to OVA+air	AHR ↑ in OVA+DE compared to OVA+air<br><br>Goblet cells ↑ in OVA+DE compared to OVA+air	Miyabara et al, 1998 [108]
Male C3H/HeN BALB/c	i.p. sensitization to OVA(+alum) or saline at day 7	5 x i.t. DEP or saline at 1-week interval and<br>OVA inhalation 4 weeks after sensitization	OVA-IgG_1↑ in C3H/HeN in OVA+DEP compared to OVA+saline<br>(no effect on OVA-IgE)	Lung eosinophils ↑ and lymphocytes ↑ in BALB/c and C3H/HeN in OVA+DEP compared to OVA+saline<br><br>Lung: IL-2 ↑ and IL-5 ↑ in C3H/HeN in OVA+DEP compared to OVA+saline	Goblet cells ↑ in C3H/HeN in OVA+DEP compared to OVA+saline<br><br>AHR ↑ in C3H/HeN in OVA+DEP compared to OVA+saline	Miyabara et al, 1998 [109]
Male ICR	i.p sensitization to OVA (+alum) or saline at week 16	34 weeks inhalation of DE or air<br>3 weeks after sensitization, 6 x OVA inhalation at 3-week intervals	No effect on OVA-IgE and IgG_1	BAL total cells ↑, macrophages ↑, neutrophils ↑, BAL and lung eosinophils ↑, lung lymphocytes ↑ in OVA/DE compared to OVA/air<br><br>Lung: IL-2 ↑ and trend to IL-5 ↑ in OVA/DE compared to OVA/air	Goblet cells ↑ in OVA/DE compared to OVA/air<br><br>Non-ciliated cell proliferation ↑ and epithelial cell hypertrophy ↑ in OVA/DE compared to OVA/air	Ichinose et al, 1998 [110]
BALB/c ICR C3H/HeN	4 x i.t. sensitization to Der f or saline at 2-week intervals at week 2	8 weeks inhalation of DE or air	Der f-IgG_1 ↑ in ICR and C3H/He in Der f+DE compared to Der f+air	Lung eosinophils ↑ (in BALB/c and ICR)<br>Lung lymphocytes ↑ (in BALB/c and C3H/He) in Der f+DE compared to Der f+air<br><br>Lung IL-3 ↓ (in BALB/c and C3H/He) lung IL-5 ↑, MCP-1 ↑, MIP-1α ↑ (in BALB/c, ICR and C3H/He) RANTES ↑ and eotaxin ↑ (in ICR and C3H/He) in Der f+DE compared to Der f+air	Increased goblet cells (in ICR and C3H/He) in Der f+DE compared to Der f+air	Ichinose et al, 2003 [111]
Male ICR	4 x i.t. sensitization to OVA or saline at 2-week intervals (no alum)	6 x i.t. DEP or saline at 1-week interval	OVA-IgG_1 ↑ in OVA+DEP compared to OVA+saline	BAL and lung polymorphonuclear cells ↑ and lung mononuclear cells ↑ in OVA+DEP compared to OVA+saline<br><br>Lung IFN-γ ↓, IL-5 ↑, IL-13 ↑, eotaxin ↑ and MIP-1α ↑ in OVA+DEP compared to OVA+saline	Goblet cells ↑ in OVA+DEP compared to OVA+saline	Yanagisawa et al, 2006 [112]

Female BALB/c	i.p. sensitization to OVA at day 1 (no alum)<br><br>i.p. sensitization to OVA(+alum) at day 1	OVA+saline or OVA+DEP inhalation at days 14-17<br><br>Or OVA inhalation at days 15-17, and saline or DEP inhalation at days 18-21	No effect on OVA-IgE and IgG_1 in OVA+DEP compared to OVA+saline	No effect on BAL cells and BAL cytokines<br><br>Inflammatory foci ↑ and major basic protein deposition ↑ in large and intermediate airways in OVA+DEP compared to OVA+saline	Mucin production ↑ in large and intermediate airways in OVA+DEP compared to OVA+saline<br><br>AHR ↑ in OVA+DEP compared to OVA+saline	Hao et al, 2003 [114]
Female BALB/c	i.p. sensitization to OVA(+alum) or saline at day 0, 6 and 7	i.n. OVA at day 21, followed by DE or air inhalation for 1 day, 1, 2, 3, 4 or 8 weeks	N.D.	No effect on inflammation cells in BAL and lung<br><br>Lung mRNA of IL-4 ↑, IL-5 ↑ and IL-13 ↑ after 1 day, MDC ↑ after 1 week and RANTES ↑ after 2 and 3 weeks in OVA/DE compared to OVA/air<br><br>BAL IL-4 after 1 day ↑ and RANTES after 8 weeks↑ in OVA/DE compared to OVA/air	No effect on goblet cells<br><br>AHR ↑ after 1 and 4 weeks in OVA/DE compared to OVA/saline	Matsumoto et al, 2006 [113]

OVA: Ovalbumin, DEP: diesel exhaust particles, Der f: Dermatophagoides farinae, BAL: Bronchoalveolar lavage fluid, OVA-Ig: OVA-specific immunoglobulin, Der f-Ig: Der f-specific immunoglobulin, AHR: airway hyperresponsiveness, i.p.: intraperitoneal, i.n.: intranasal, i.t: intratracheal, LN: lymph node, N.D.: not determined
